# Marginal Stability of the YB1 Cold‐Shock Domain in Cells Enables Binding of Multiple Nucleic Acids

**DOI:** 10.1002/advs.202512966

**Published:** 2025-10-27

**Authors:** Puja Shrestha, Sara S. Ribeiro, Janne Aurich, Christian Herrmann, Simon Ebbinghaus

**Affiliations:** ^1^ Lehrstuhl für Biophysikalische Chemie and Research Center Chemical Sciences and Sustainability Research Alliance Ruhr Ruhr University Bochum Universitätsstraße 150 44801 Bochum Germany; ^2^ Institute of Physical and Theoretical Chemistry Technical University Braunschweig Rebenring 56 38106 Braunschweig Germany; ^3^ Department of Physical Chemistry I Ruhr University Bochum Universitätsstraße 150 44801 Bochum Germany

**Keywords:** folding and binding free‐energy landscapes, in‐cell protein folding, nucleic acid binding, YB1‐cold shock domain

## Abstract

YB1 is an intrinsically disordered protein with a folded cold shock domain (CSD) required for translation, transcription, and RNA metabolism. This multifunctionality and cancer involvement make it a therapeutically attractive target. YB1‐CSD and nucleic acid interaction is essential for function. The CSD is marginally stable in vitro, with unknown implications for its function in the cell. In this study, the folding stability of the CSD in living cells is studied at the physiological levels of nucleic acids. The CSD is highly stabilized (increase in T_M_ by 10 °C) by a disordered 11 amino acid tail encoded in the C‐terminal YB1‐domain (CSDex). Still, CSDex remains 50% folded and 50% unfolded inside cells, whereas CSD alone is mostly unfolded. The stability strongly increased upon nucleic acid binding, an effect that is sequence‐dependent and stronger for RNA than DNA. The thermodynamic and kinetic analysis revealed an ensemble of native state conformers for CSDex when probed in its native cellular environments. It is proposed that this allows the protein to retain its native fold upon binding to different ligands. The marginal stability can be a trade‐off for its high demand to bind efficiently to different ligands, mediating the multifunctionality of YB1.

## Graphical abstract

This study suggests that the marginal stability of the YB1 Cold‐Shock Domain (CSDex) allows for a balance between multifunctionality and conformational changes under physiological conditions. This balance is driven by the dynamic equilibrium between the unfolded ensemble (U_E_) and the native ensemble (N_E_) of CSDex (PDB:6LMR). This N_E_ comprises multiple conformers enabling binding to a diverse range of ligands (N_B_) in living cells.

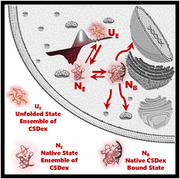

## 1 Introduction

Initially known as p50 protein, Y‐Box binding protein 1 (YBP1/YB1) is a well‐studied intrinsically disordered protein (IDP) with an evolutionary conserved folded DNA/RNA binding domain.^[^ ^1^, ^2^, ^3^, ^4^, ^5^ ^]^ In 1988, YB1 was found to bind the Y‐Box sequence (dsDNA: CCAAT/ATTGG) in the promoter region of the major histocompatibility complex class (MHC) II.^[^ ^6^ ^]^ Several follow‐up studies showed that it is not only specific to the MHC II but it can also bind the enhancer region of the epidermal growth factor receptor (EGFR) gene^[^ ^7^ ^]^ and multidrug resistance 1 (MDR1).^[^ ^8^ ^]^ This multifunctionality is important in cellular processes like translation, transcription, cell replication, embryonic development, and stress granule formation.^[^ ^9^, ^10^, ^11^, ^12^, ^13^ ^]^ In addition, YB1 was shown to activate the transcription of several “proliferative” genes in cancer cells of different genesis upon binding to promoter regions.^[^ ^14^ ^]^ With this, YB1 hyperphosphorylation, causing nuclear translocation, has been correlated with various cancers, resulting in poor prognosis.^[^ ^15^, ^16^, ^17^, ^18^, ^19^, ^20^ ^]^ Further, it is known to protect some cancer cells against apoptosis^[^ ^21^, ^22^ ^]^ through, for instance, modulation of p53 activity.^[^ ^23^ ^]^ Therefore, YB1 has been suggested to be an important cancer marker^[^ ^24^ ^]^ and a potential target for cancer therapy.^[^ ^9^, ^15^, ^16^, ^25^ ^]^ Additionally, it was suggested to be a therapeutic target for HIV, as a study by Weydert et al. demonstrated that YB1 is a cofactor and supports early and late‐step HIV replication. By depleting YB1 expression, a 10‐fold decrease in HIV‐1 replication was observed in different cell lines, thus being a promising candidate for drug targeting.^[^ ^26^ ^]^

YB1 consists of three domains: the N‐terminal A/P domain (residues 1–51, rich in alanine/proline), the cold shock domain (CSD, residues 52–129, highly conserved and structured region), and a highly charged and disordered C‐terminal domain (CTD, residues 130–324).^[^ ^4^, ^6^, ^9^ ^]^ The CSD is known to bind nucleic acids, single or double‐stranded DNA and RNA. Most of the YB1 functions are associated with the binding of ligands (DNA/RNA) to CSD.^[^ ^27^, ^28^, ^29^, ^30^ ^]^ Surprisingly, this domain is folded into an anti‐parallel ß‐barrel structure, but only marginally stable. Thermal melting experiments yielded a melting temperature of (T_M_ = 41 °C) under dilute buffer conditions.^[^ ^31^ ^]^ This raises the intriguing question if the CSD domain is folded or intrinsically disordered under physiological conditions.

The marginal stability could in part be caused by the unfavorable surface exposure of conserved nonpolar aromatic residues^[^ ^32^, ^33^, ^34^ ^]^ in the CSD (Figure S1, Supporting Information), essential for DNA/RNA binding.^[^ ^35^, ^36^ ^]^ This could be due to an evolutionary pressure on the protein to balance function and stability, where due to the gain of function, stability is compromised.^[^ ^37^, ^38^, ^39^, ^40^, ^41^, ^42^, ^43^ ^]^ This was demonstrated in a study by Wang and coworkers.^[^ ^5^ ^]^ The authors modified the bacterial cold shock protein A (CspA) by replacing a short six–amino acid loop (loop 34, between strands β3 and β4) with the equivalent loop from the human protein YB1‐CSD. This hybrid CspA was then able to bind to double‐stranded nucleic acids, but it also made the protein less stable (hybrid CspA T_M_ = 50 °C and CspA T_M_ = 60 °C). Loop 34 in human YB1 is longer than loop 34 of CspA,^[^ ^5^ ^]^ suggesting that the alteration of the loop length could be a mean of evolutionarily modulating function and stability.^[^ ^44^ ^]^

Since the conformational dynamics of the CSD in cells are unclear, the mechanism by which it interacts with different nucleic acids remains elusive. In general, ligand binding to proteins is often described by two mechanisms: conformational selection and induced fit. In conformational selection, the ligand preferentially binds to a preexisting, low‐populated conformer of the protein, stabilizing it in the bound state. In contrast, the induced fit model postulates that the ligand initially binds to the predominant free conformation of the protein, subsequently inducing structural rearrangements that lead to the final bound state.^[^ ^45^, ^46^, ^47^, ^48^ ^]^ In the context of protein‐protein (e.g., heat‐shock protein 70 (HSP70)–substrate)^[^ ^49^ ^]^ or protein‐nucleic acid interactions (e.g.,  *lac* repressor headpiece–DNA),^[^ ^50^ ^]^ conformational selection is proposed as the predominant molecular recognition mechanism.^[^ ^51^ ^]^

Coupled with the binding mechanism are the alterations in the protein folding free‐energy landscape upon interaction with ligands, reflecting the stabilization of specific conformational states. Preferential binding of ligands to unfolded and partially folded states generally destabilizes the protein while suppressing aggregation.^[^ ^52^ ^]^ Preferential binding of ligands to the folded state results in stabilization via Le Chatelier's principle.^[^ ^53^, ^54^, ^55^ ^]^ Some ligands can also actively remodel the protein folding free‐energy landscape by binding and assisting the folding of unfolded or partially folded states in a process known as “folding upon binding.”^[^ ^56^, ^57^ ^]^ This latter has been extensively studied in the context of ligand‐driven regulation of IDPs.^[^ ^56^, ^58^, ^59^ ^]^ Proteins capable of binding multiple ligands, such as myoglobin and bovine serum albumin, typically exist as a dynamic ensemble of isoenergetic native conformations, with the relative populations of these conformers modulated by ligand type and concentration.^[^ ^60^, ^61^ ^]^ For CSD, the extent to which nucleic acids remodel their folding landscape remains largely unexplored. Given CSD's capacity to bind multiple nucleic acids in its native state, it is plausible that its folding landscape is more complex than a simple two‐state model.

In this study, we investigate the folding stability and kinetics of the CSD extend by a disordered 11 amino acid tail encoded in the C‐terminal YB1‐domain (CSDex) as a function of ligand binding in living cells and by complementary in vitro experiments. The in‐cell folding experiments allow us to study the protein in its physiological environments under different conditions, accounting for the multiplicity of ligands at native concentrations. The in vitro folding and binding experiments, comprising isothermal titration calorimetry (ITC), circular dichroism (CD) spectroscopy, and fluorescence microscopy, allow us to disentangle the effects of different concentrations and sequences of DNA/RNA. Studying CSDex, we found that the C11‐tail has an additional nucleic acid binding site crucial for stability. We could show that the protein is still marginally stable in cells, sampling different conformations in its native state that are tuned for specific ligand interactions.

## 2 Results

### 2.1 CSD Folding Studies in Cells by Fast Relaxation Imaging

**Figure 1:**
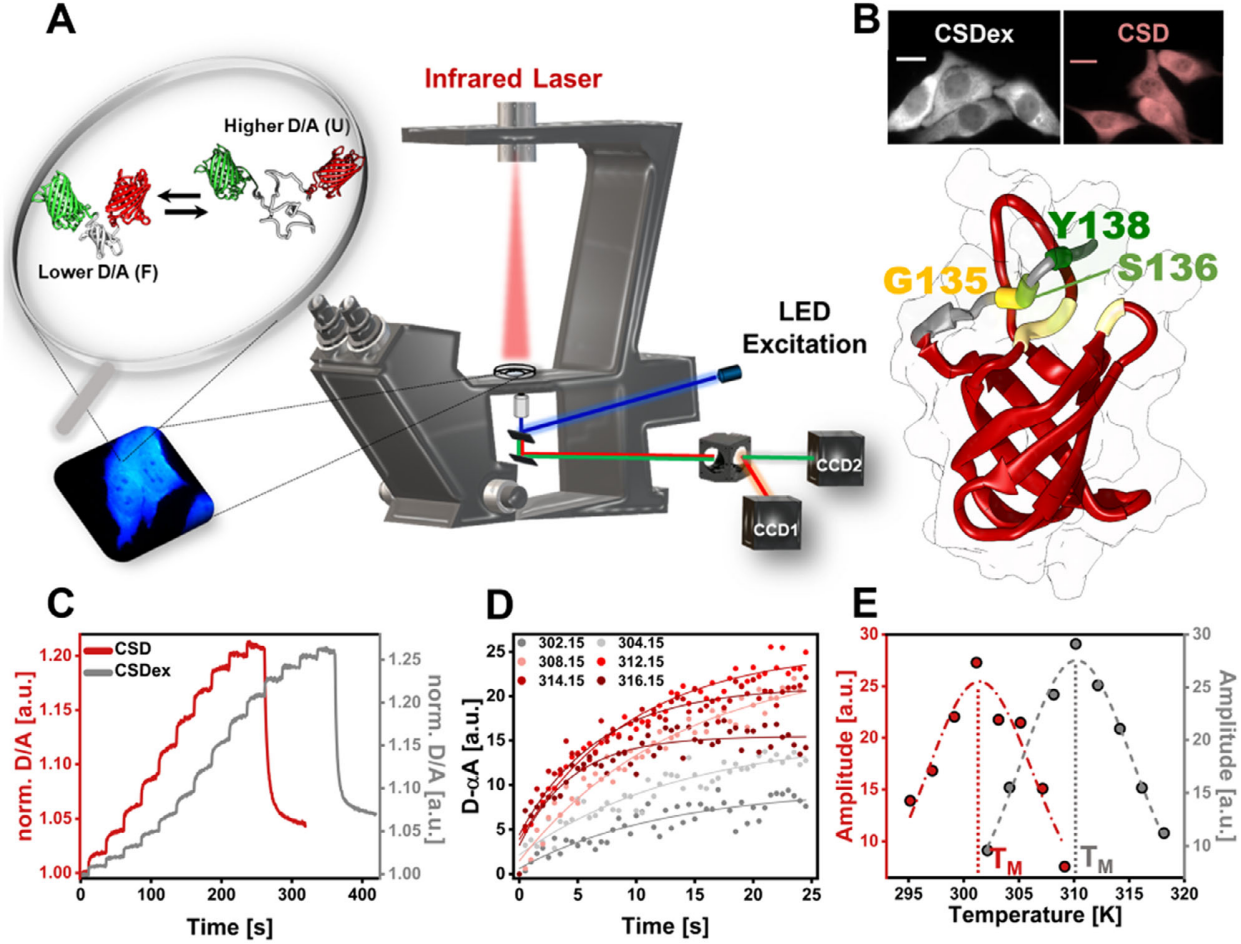
Fast relaxation imaging (FReI) to investigate YB1‐CSD folding stability in‐cell. A) FReI setup. The inset shows the equilibrium between folded and unfolded states of AcGFP1‐CSD‐mCherry transfected into HeLa cells. B) Structure of CSD of YB1 human protein (PDB: 6LMS): CSD (52–129) is presented in red while CSD extended by the disorder C11‐tail (CSDex) is presented in grey. Scale bar stands for 20 µm. C) Normalised FRET (D/A) ratio as a function of time showing a heat‐induced unfolding FReI curve of CSD and CSDex in HeLa cells. D) Exemplary relaxation kinetics (D‐αA) of CSD unfolding upon temperature increase. A single exponential model was used to fit the single temperature jumps to determine the kinetic unfolding amplitude (D‐αA vs. T). E) Representative kinetic amplitudes as a function of temperature to determine T_M_ for CSD and CSDex.

We used Fast relaxation imaging (FReI)^[^ ^62^ ^]^ to quantify the CSD and folding stability in cells and in vitro (**Figure** **1**A). Therefore, we attached two fluorophores, AcGFP1 (Donor [D]) and mCherry (Acceptor [A]), to the N‐ and C‐terminus of the CSD (Figure 1A, inset). The CSD of YB1 is composed of a five‐stranded anti‐parallel ß‐barrel and a lengthy flexible loop between the ß3 and ß4 strands (Figure 1B). In addition, we chose to study a CSD construct encompassing the C11 tail, named CSDex, from the CTD of YB1. NMR studies identified functional interactions between the tail and the backbone of the CSD G135‐V103, G135‐G104, S136‐S102, and Y138‐V68. An additional binding site for nucleic acids was further proposed involving Y138 located within the C11 tail^[^ ^63^ ^]^ (Figure 1B). It is of note that in some experiments the CSD (without the CTD tail), named CSD, is studied for comparison.

Transfected cells or in vitro samples were heated via fast‐temperature jumps (10–18, 25 s each) in the millisecond range using a mid‐infrared (IR) laser (2200 nm) which is customized into a wide‐field microscope (Figure 1A; Figure S2, Supporting Information).^[^ ^62^ ^]^ The fast temperature jumps cause the thermal unfolding of FRET‐CSDex, assessed by a decrease in the FRET signal (higher D/A) as the heating progresses in time (Figure 1C). Unfolding kinetic amplitudes at each temperature were scaled (D‐αA; α = D_0_/A_0_) and fitted with single or stretched exponential models in cell (Figure 1D) or in vitro, respectively, to determine the amplitude at the steady state. The derived amplitudes were further plotted against temperature and fitted with a two‐state folding model to determine T_M_ (Figure 1E) and cooperativity parameter δg1. δg1 represents the prefactor of the linear Taylor approximation of the two‐state population, and T_M_ denotes the melting point associated with the protein unfolding transition, where half of the protein population is unfolded and half remains folded. The modified standard state free energies of folding (ΔG_F_^0’^[37 °C]) were calculated via the equation; ΔG_F_^0’^(37 °C) = δg1 * (T − T_M_), T = 310 K or 37 °C.^[^ ^64^ ^]^ ΔG_F_^0’^ is related to the folding equilibrium constant (K_F_) by: ΔG_F_^0’^ = −R*T * lnK_F_. ΔG_F_^0’^ quantifies how thermodynamically favorable the folded state of a protein is compared to the unfolded state under physiological conditions. A large, negative ΔG_F_^0’^ means folding is strongly favored, and most proteins will be in the folded state. Although changes in ΔG_F_^0’^ can be small and less than the thermal energy, these changes can still cause decisive shifts in population with a major impact on biological systems.

To verify that the FRET pair did not interfere with CSDex folding stability, we monitored the thermal unfolding of purified FRET‐labeled and unlabeled protein (Figure S3A,B, Supporting Information) using temperature‐dependent circular dichroism (CD) spectroscopy. BeStSel‐predicted secondary structure^[^ ^65^ ^]^ for native, unlabeled CSDex closely matches that of the corresponding PDB structure (6LMS), supporting correct folding at 20 °C (Figure S3C,D, Supporting Information). We further confirmed that the thermal unfolding was reversible (Figure S3E–G and Table S1, Supporting Information). Folding stabilities determined from CD‐thermal unfolding curves for both FRET‐labeled and unlabeled‐CSDex were in close agreement (T_M_^CD‐FRET^ = 315.9 ± 2.5 K, T_M_^CD‐unlabeled^ = 315.3 ± 0.9 K; Figure S3H, Supporting Information), demonstrating that the presence of fluorophores does not alter the folding stability in vitro. These findings are consistent with previous studies showing that labeling with the FRET pair AcGFP1/mCherry does not perturb the folding stability of various globular proteins.^[^ ^62^, ^66^, ^67^, ^68^ ^]^

### 2.2 The CSD Is Unstable Inside the Cell While CSDex Is Marginally Stable

**Figure 2:**
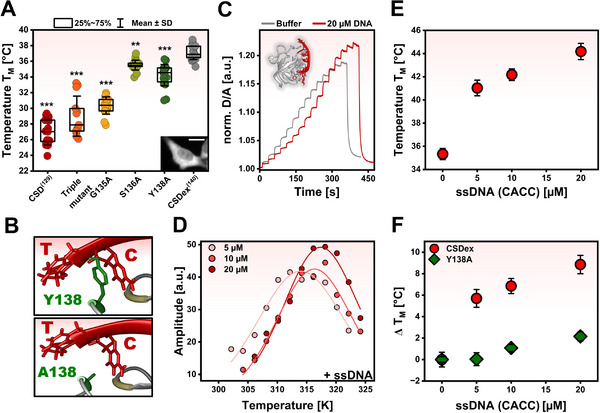
Folding stabilities of CSD and CSDex in‐cell and in vitro. A) In‐cell: T_M_ of CSD and CSDex with mutations G135A, S136A, Y138A, and triple mutant. Statistical differences between CSDex and the other constructs are shown (^**^ *p* < 0.01, ^***^ *p* < 0.001). Scale bar 20 µm. B) Nucleic acid‐CSDex Y138 interaction (top) and mutational effect (bottom; PDB: 6LMR). C) In vitro: Heat‐induced unfolding FReI curve of purified 5 µM CSDex in buffer and in the presence of 20 µM DNA. D) Kinetic amplitudes as a function of temperature to determine T_M_ for CSDex (5 µM) and in the presence of DNA (5, 10, and 20 µM). E) T_M_ of CSDex (5 µM) in the presence of DNA (CACC; mean ± SEM). F) ΔT_M_ for CSDex and CSDex‐Y138A against different concentrations of DNA (mean ± propagated SEM; ΔT_M_ = T_M_^LC^‐T_M_^Buffer^ [LC‐added ligand condition, buffer condition]).

We first studied the folding stability of CSD and CSDex inside HeLa cells by FReI. We found that T_M_ is 27.0 ± 2 °C for CSD and 37.1 ± 0.9 °C for CSDex (**Figure** **2**A). This result is interesting in two ways. First, we find that as predicted by in vitro experiments,^[^ ^31^, ^63^, ^69^, ^70^ ^]^ CSDex is indeed marginally stable in cells (≈50% folded and ≈50% unfolded) and not significantly stabilized under native conditions. Second, we find that the interactions between the C11‐tail and the CSD, as suggested by Zhang et al.,^[^ ^63^ ^]^ are crucial for protein folding stability, changing the melting temperature by 10 °C and the population of folded protein from ≈5% to ≈50% (Figure S4, Supporting Information). Two possible explanations behind the increased folding stability of CSDex compared to CSD according to the previous NMR studies^[^ ^63^ ^]^ are: (I) the disordered C11‐tail from the CSDex interact with the residues from CSD so that it gains structural stability and (II) the Y amino acid at position 138 provides an additional interaction partner with nucleic acids. To explore the first possibility, an alanine single‐point mutation, Y138A, S136A, and G135A, and one triple mutation (Y138A, S136A, and G135A) were investigated. Mutations S136A and Y138A decrease the T_M_ of CSDex by 1.6 and 2.8 °C, respectively, while G135A decreases CSDex T_M_ by 6.7 °C (Figure 2A). Thus, G135, S136, and Y138 interactions increase the stability of CSDex, with the largest contribution of G135. As expected, the triple mutant also decreased T_M_ by 8.1 °C (Figure 2A). Nevertheless, this triple mutant still shows a higher T_M_ (≈2 °C) compared to the CSD, suggesting that these three residues are not the only determinants of CSDex folding stability. Indeed, recent MD simulations showed additional residues involved in the structural stability of the latter.^[^ ^71^ ^]^

We next test the second hypothesis and investigate if the decrease in stability for the Y138A mutation is due to the loss of interaction partner to nucleic acids (Figure 2B). To test the impact of this mutant on folding stability and how the latter might be modulated by nucleic acid binding, we purified CSDex and its mutant Y138A and determined its folding stability in the absence and presence of added DNA. We tested ssDNA 5′ aaCACCt 3′ at three different concentrations (5, 10, and 20 µM) as previous studies reported strong binding of CSDex with CACC sequence.^[^ ^63^ ^]^ An increasing concentration of DNA shows an increase in the folding stability (Figure 2C,D), with a rise of ≈9 °C in T_M_ (Figure 2E) and a decrease of ≈4 kJ/mol in ΔG_F_^0’^ (37 °C) upon the addition of 20 µM DNA (Figure S5A, Supporting Information). Thus, CSDex folding stability is significantly higher upon DNA binding.

In contrast, we observed an increase of ≈2 °C and a decrease of 0.7 kJ/mol on Y138A T_M_ and ΔG_F_^0’^ (37 °C), respectively, after adding 20 µM DNA (Figure S5B,C, Supporting Information). The observed changes in Y138A are significantly smaller than in CSDex (Figure 2F), suggesting that Y138 is important for binding to nucleic acids.

In summary, the disordered C11‐tail in CSDex stabilizes the protein by 10 °C in T_M_ or ≈8 kJ mol in ΔG_F_^0’^ compared to the CSD lacking the tail in‐cell. We could show that the interaction between the residues S136, G135, Y138, and CSD backbone was responsible for this enhancement, with Y138 influencing both folding and ligand binding. Yet, CSDex still remains marginally stable under physiological conditions.

### 2.3 Ligand and Sequence‐Dependent Stabilization of CSDex

**Figure 3:**
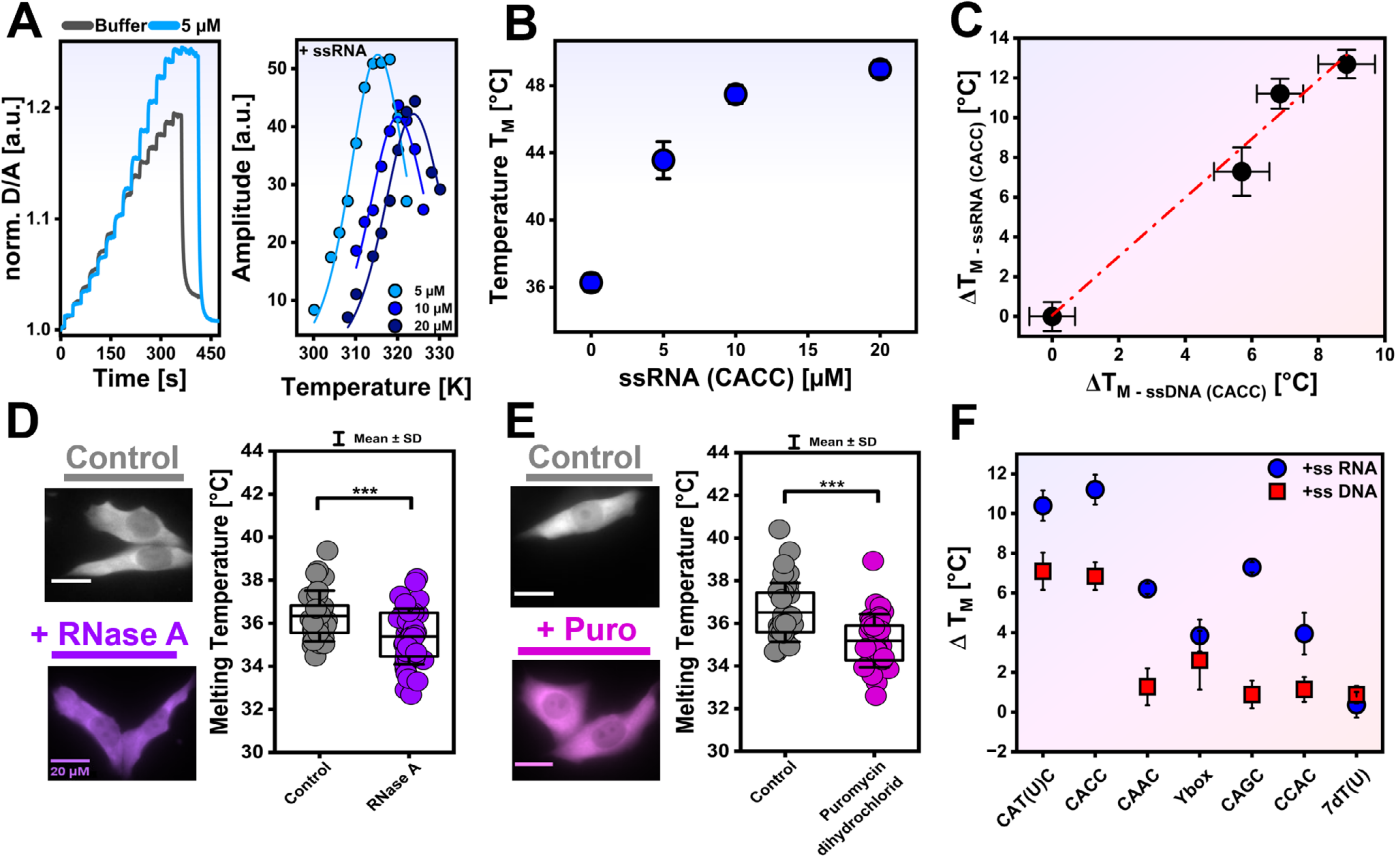
Effect of nucleic acids on CSDex folding stability. A) In vitro: Heat‐induced unfolding FReI curve of purified protein (5 µM) and upon addition of 5 µM RNA (left). Kinetic amplitudes as a function of temperature to determine T_M_ in the presence of 5, 10, and 20 µM of RNA (right). B) T_M_ in the presence of RNA (CACC at 5, 10, and 20 µM; mean ± SEM). C) ΔT_M_ of CSDex at different concentrations of DNA on the x‐axis and RNA on the y‐axis. The slope of the linear relationship is 1.48 (*R* ^2^ = 0.98; mean ± propagated SEM). D) In‐cell: T_M_ in cells treated with puromycin hydrochloride (100 µg/mL) for 30 min. E) In‐cell: T_M_ in cells treated with RNase A (5 µg/mL) for 1 h (^***^ *p* < 0.001). F) In vitro: ΔT_M_ for CSDex in the presence of DNA and RNA with different sequences (mean ± propagated SEM; ΔT_M_ = T_M_^LC^‐T_M_^Buffer^ [LC stands for added ligand]).

The ability of CSDex to interact with DNA via the C11‐tail and its subsequent stabilization raises the question of how RNA interaction tunes its folding stability. Given that the YB1 is predominantly localized in the cytoplasm, where RNA is abundant, we examined how RNA modulates CSDex folding stability under physiologically relevant conditions. Hence, we investigated the impact of RNA on the folding stability of the protein in vitro and inside cells. First, we used the ssRNA counterpart of the ssDNA 5′ aaCACCt 3′, with the sequence 5′ aaCACCu 3′, and measured the folding stability of CSDex in the presence of 5 µM, 10 µM, and 20 µM of RNA (CACC; **Figure** **3**A) in vitro. The addition of RNA showed a nonlinear increase in T_M_ up to 13 °C (Figure 3B) and a decrease in ΔG_F_^0’^(37 °C) up to 7 kJ/mol at the highest concentration tested (Figure S5D, Supporting Information). Interestingly, the increase in folding stability with RNA is by a factor of ≈1.5 higher than for DNA (Figure 3C), showing that RNA binding stabilises the CSDex more strongly than the DNA. Second, we reduced the RNA content in cells and measured the folding stability upon RNA depletion. We treated cells with puromycin dihydrochloride (Figure 3D) and RNase A (Figure 3E), both of which are known to decrease RNA cellular content.^[^ ^72^, ^73^, ^74^, ^75^ ^]^ Indeed, both treatments led to a significant reduction of T_M_ (Figure 3D,E), showing that CSDex folding stability in‐cell is tightly regulated by RNA binding.

YB1 multifunctional roles are linked to its interactions with DNA and RNA.^[^ ^10^, ^26^, ^30^, ^63^, ^76^, ^77^, ^78^, ^79^, ^80^ ^]^ We further asked whether DNA or RNA sequences with different functions induce distinct changes in CSDex folding stability, and whether the greater stabilizing impact of RNA versus DNA is observed across sequences. Therefore, we compared different DNA and RNA sequences (Table S2, Supporting Information) at a concentration of 10 µM. Several of these DNA sequences are found in regions that initiate or inhibit transcription, such as the multidrug‐resistant 1 (MDR1) gene promoter containing CACC and CAGC,^[^ ^8^ ^]^ EGFR containing CAAC and CAGC,^[^ ^7^ ^]^ MYC gene containing CACC,^[^ ^81^ ^]^ MHCII and p53 promoter containing Y‐box sequence.^[^ ^6^, ^82^ ^]^ The RNA counterparts could also be important for CSDex regulation of translation of multiple proteins (ferritin,^[^ ^83^ ^]^ HSP70,^[^ ^84^ ^]^ G3BP1,^[^ ^85^ ^]^ and YB1 auto‐regulation^[^ ^86^ ^]^), for example, the translation of mRNA CD44 v5 exon, containing CAUC.^[^ ^87^ ^]^ We observed an increase in the CSDex folding stability for the addition of DNA, for the sequences CATC, CACC, and Ybox, with minor effects for CAGC, CCAC, CAAC, and 7dT used as a nonspecific control (Figure 3F). In line with the previous experiments, we observed a stronger stabilization for RNA that is evident for all sequences studied (7dU used as a control; Figure 3F).

Overall, the experiments show that RNA binding increases CSDex folding stability inside cells and in vitro. Further, the results reveal that the magnitude of stabilisation upon binding to RNA is higher than upon binding to DNA in a sequence‐dependent manner.

### 2.4 Mechanism of CSDex‐Nucleic Acid Binding

**Figure 4:**
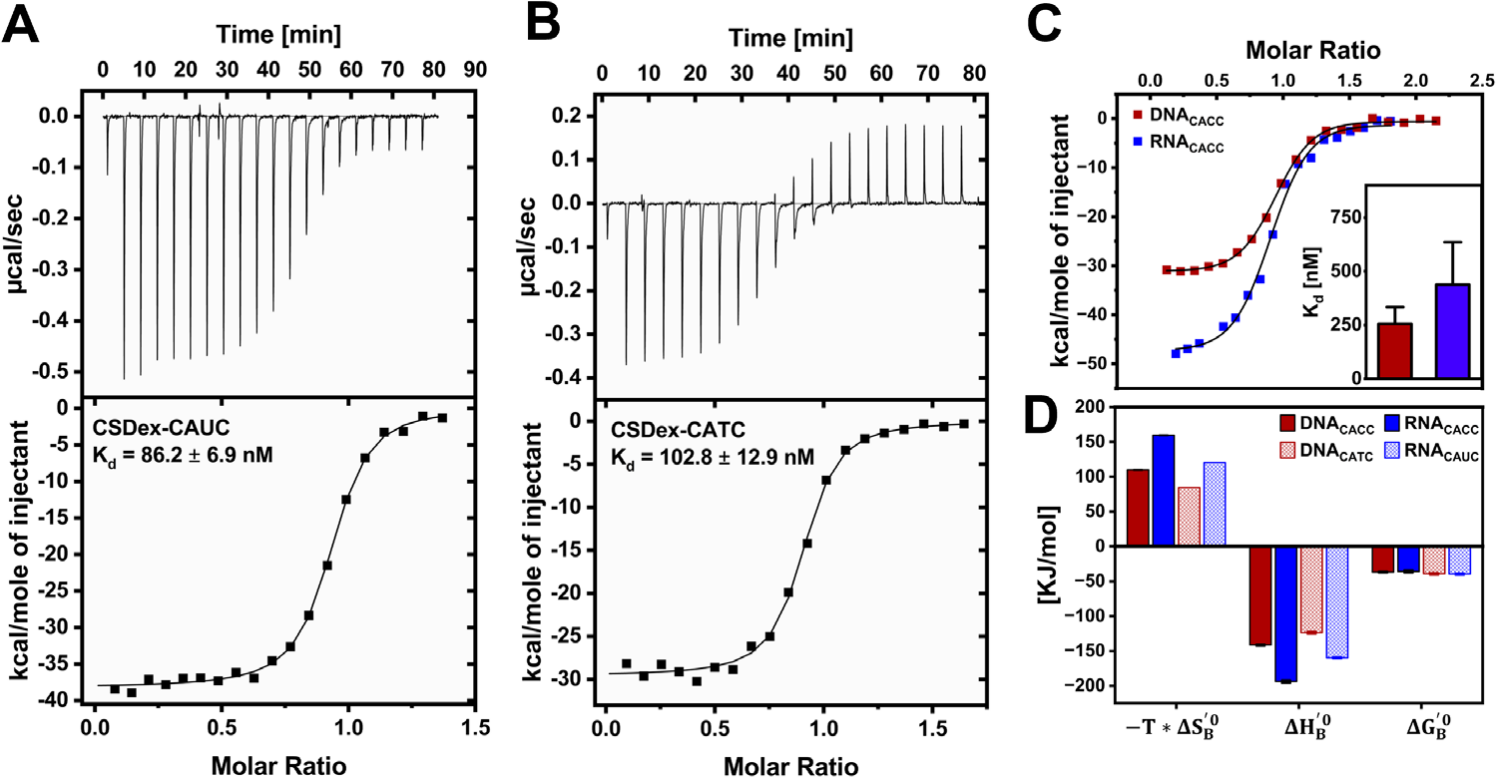
ITC profiles of CSDex binding to different nucleic acids: A) RNA (CAUC) and B) DNA (CATC). K_d_ ± fitting error in (A) and (B). C) CACC DNA (red) and RNA (Blue). The inset shows weighted means ± SDs. D) Thermodynamic parameters of binding in kJ/mol.

According to previous studies, CSD exhibits a stronger binding affinity for RNA than for DNA.^[^ ^10^, ^80^, ^88^ ^]^ We therefore performed ITC and CD spectroscopy to uncover the mechanisms behind CSDex‐nucleic acid interaction and to know whether binding affinity drives these changes and shapes its multifunctional activity. The ITC measurement was carried out with the two stronger stabilising sequences, CATC and CACC, in both DNA and RNA, at 20 °C (**Figure** **4**A–C). The ITC results revealed that binding is exothermic, with affinities up to several hundred nanomolar. The dissociation constant (K_d_) for the CSDex with RNA_CAUC_ was 86 ± 7 nM, whereas for DNA_CATC_, it was 102 ± 13 nM (Figure 4A,B). Likewise, the K_d_ values for CSDex with DNA_CACC_ and RNA_CACC_ were 250 ± 14 nM and 380 ± 30 nM (Figure 4C), respectively. These results show that CSDex binds RNA and DNA with similar affinities. Thus, the greater stabilisation upon RNA binding, relative to its DNA counterpart, is not driven by differences in binding affinity. The results rather suggest that the protein adapts different conformers when bound to DNA versus RNA.

To investigate the molecular driving forces underlying CSDex binding to DNA and RNA, we compared the binding enthalpy (ΔH_B_^0’^) and entropy (ΔS_B_^0’^) contributions for DNA and RNA interactions derived from ITC data. The thermodynamic analysis showed that the binding is enthalpy‐driven, counteracted by a significant entropic cost (Figure 4D). Notably, the CSDex‐RNA complex exhibits more favorable binding enthalpies and more unfavorable binding entropies compared to the corresponding DNA complex (Figure 4D), indicating different interaction patterns or solvation effects upon binding.^[^ ^89^, ^90^, ^91^ ^]^ To investigate if DNA and RNA bound conformers show distinct secondary structure, we recorded CD spectra of CSDex in the presence of DNA (CACC) or RNA (CACC) at 20 °C with a 2:1 protein‐to‐nucleic acid ratio (Figure S6A,B, Supporting Information). DNA and RNA binding induced distinct changes in CSDex secondary structure (Figure S6C,D, Supporting Information), indicating different folded states with each nucleic acid.

Together, our results demonstrate that the observed stability enhancement cannot be explained by binding affinity alone. Instead, it arises from different CSDex conformations and interaction patterns with DNA versus RNA. These findings show the conformational diversity underlying CSDex‐DNA or RNA interactions with functional implications.

### 2.5 Folding Studies Report on the Mechanism of CSDex Stabilization

**Figure 5:**
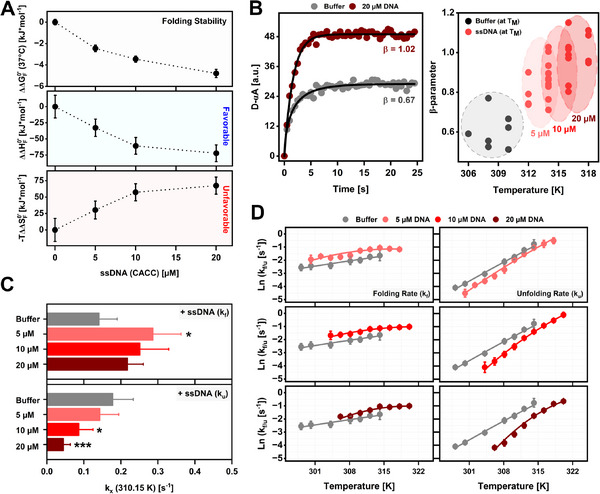
Modulation of CSDex folding by DNA/RNA binding in vitro. A) Enthalpic changes (ΔΔH_F_^0’^), entropic changes (−TΔΔS_F_^0’^), and folding free energy changes (ΔΔG_F_^0’^[37 °C]) for CSDex (5 µM) in buffer and the presence of 5, 10, and 20 µM DNA (mean ± propagated SEM). B) Left: Exemplary relaxation kinetics (D‐αA) of CSDex unfolding at T ≈ T_M_ in buffer and 20 µM DNA. Right: ß‐parameter for CSDex in buffer condition and CSDex + DNA at their corresponding ≈T_M_. C) CSDex folding (k_f_) and unfolding (k_u_) rate constants at 37 °C in the absence and presence of 5, 10, and 20 µM DNA (mean ± SD; ^*^ *p* < 0.05, ^***^ *p* < 0.001). D)Temperature‐dependent folding (k_f_) and unfolding (k_u_) rate constant plots for CSDex before and after addition of DNA (mean ± SEM). Solid lines are fits to an effective two‐state rate model (Equation 14). Resulting parameters are shown in Table S3 (Supporting Information).

By performing in‐cell protein stability measurements on a set of different variants and mutants of CSDex (section 2.2) and complementary ligand binding assays in vitro (section 2.3), we could show that nucleic acid binding determines the folding stability of CSDex, which is marginally stable in cells. In this section, we analyse the factors governing this folding stability, including the underlying thermodynamic driving forces as well as the folding and unfolding kinetics. To analyse the thermodynamic driving forces, we dissect the ΔG_F_^0’^ into its enthalpic (ΔH_F_^0’^) and entropic (ΔS_F_^0’^) components using Gibbs’ equation:^[^ ^92^, ^93^ ^]^ ΔG_F_^0’^ = ΔH_F_^0’^ − T*ΔS_F_^0’^. The thermodynamic folding parameters X = G, H, S are expressed as changes between the ligand‐bound (LC) and the buffer conditions: ΔΔX_F_^0’^ = ΔX_F_^0’LC^ − ΔX_F_^0’Buffer^. For the stabilization of CSDex by increasing concentrations of CACC DNA (ΔΔG_F_^0’^(37 °C) < 0), we found an increasingly favorable folding enthalpy (ΔΔH_F_^0’^< 0) and an unfavorable folding entropy (−TΔΔS_F_^0’^ > 0; **Figure** **5**A). This shows that the stabilisation upon nucleic acid binding to the native state is an enthalpy‐driven process. In a similar way, the addition of RNA (CACC) also induced an enthalpic stabilisation of CSDex folding at all concentrations investigated (Figure S7A, Supporting Information). Furthermore, we observed a strong decrease in the initial D/A with increasing RNA concentration (Figure S7B, Supporting Information), indicating a more compact native conformation upon RNA binding. Both findings support a nucleic acid stabilization mechanism in which nucleic acid binds and strengthens the intramolecular interactions in CSDex native state, leading to a more compact conformation of the protein.

Further mechanistic insights into DNA‐mediated stabilization of CSDex are obtained by analysing the folding and unfolding kinetics that constitute CSDex stability. Observed rate constants (k_obs_) for each temperature were determined from stretched exponential fits to the unfolding kinetic amplitudes (Figure S8A, Supporting Information). Stretched exponential kinetics fit time‐dependent processes that are heterogeneous, often due to multiple overlapping pathways instead of a single rate‐limiting step (single exponentials). The stretched exponential function is exp[−(t/τ)^β^] where 0 < β≤ 1; for β = 1, it reduces to a standard single exponential. The exponent β < 1 captures a broad distribution of relaxation times rather than a single time constant. The fitted “rate constant,” τ, is a fit parameter representing a mean or characteristic relaxation time, and β indicates the degree of heterogeneity, with a smaller value revealing a more complex folding mechanism.^[^ ^94^ ^]^ Our measurements showed that the unfolding kinetics of CSDex yielded a lower ß‐parameter (0.6 ± 0.1 (mean ± SD) at T_M_) in buffer. In the presence of DNA, unfolding becomes almost single exponential (0.9 ± 0.11 [mean ± SD] at T_M_; Figure 5B). This shows that DNA binding smoothens the folding landscape compared to the unbound unfolding process. In other words, the ligand reduces the conformational space that is sampled upon CSDex unfolding, in agreement with the thermodynamic view of enthalpic compaction of the folded state.

Finally, we analysed the temperature‐dependency of the folding kinetics to elucidate the mechanism by which DNA modulates CSDex folding. The folding (k_f_) and unfolding (k_u_) rate constants at the different temperatures were calculated by applying a two‐state model for the equilibrium constant of folding (K_F_) and the rate constants (k_obs_, k_f_, k_u_): K_F_ = k_f_/k_u_ and k_obs_ = k_f_ + k_u_ (Equations 12 and 13). The two‐state assumption is supported by previous work showing that stretched folding kinetics can be described by an effective two‐state model.^[^ ^95^ ^]^ At 37 °C, DNA addition significantly increased the folding rate constant (k_f_) and significantly decreased the unfolding rate constant (k_u_; Figure 5C; Figure S8B, Supporting Information). Importantly, CSDex unfolding was progressively slowed as DNA concentration increased, consistent with stronger DNA binding and the resulting stabilization of the native state. Conversely, folding was fastest at 5 µM, while higher concentrations led to slightly reduced rates (Figure 5C). This implies that the ligand remained weakly bound to the unfolded conformers of CSDex. Similar, slower unfolding and faster folding upon ligand binding was previously observed for α‐lactalbumin: Ca^2+^ interaction.^[^ ^96^ ^]^

We noticed that increasing the concentration of DNA led to a curvature in the temperature‐dependent folding and unfolding rate plots (Figure 5D). This explains why the unfolding rate decreases more markedly, while the folding rate shows only modest acceleration at lower temperatures. This behavior likely arises from thermodynamic stabilization of CSDex‐DNA bound states at higher DNA concentrations. Binding to the native state slows unfolding, while binding to the unfolded state leads to a plateau in the folding rate as the temperature decreases. A similar mechanism was observed for the pharmacological chaperone Fe‐TMPyP binding to prion protein PrP.^[^ ^97^ ^]^

Lastly, we tested whether RNA binding induced similar changes in folding kinetics. Upon RNA addition, we found ß‐parameters for CSDex (un)folding to be significantly higher in the presence of RNA (0.9 ± 0.12 [mean ± SD]) compared to its absence (0.6 ± 0.10 [mean ± SD]; Figure S9A, Supporting Information). Further, we observed an increase in the folding rate (k_f_) and a decrease in the unfolded rate (k_u_) across all temperatures (Figure S9B, Supporting Information). Together, these findings show that RNA binds to the native state, while accelerating and smoothing the folding process. Thus, DNA and RNA binding modulate CSDex folding landscape through similar mechanisms.

In summary, DNA and RNA preferentially bind to the CSDex native state, stabilising it enthalpically and thereby slowing unfolding.

## 3 Discussion

### 3.1 CSDex Mutation, Ligand Variation, and the Impact on Folding Stability

CSDex, encompassing the C11‐tail, showed a higher in‐cell folding stability with an increase of T_M_ by 10 °C compared to the CSD alone. Mutational analysis showed that G135, S136, and Y138 each contribute to this stabilisation, with G135 providing the dominant effect (Figure 2A). G135 interacts with V103 and G104 residues, while S136 interacts with S102 within loop 34. These contacts rigidify the loop, yielding a more stable conformation. Importantly, Y138 contributes to native‐state stabilisation through nucleic acid binding, the latter being by π–π stacking between Y138 and C6^[^ ^63^ ^]^ from DNA.

Our in vitro results showed an enhanced CSDex folding stability upon binding with DNA and RNA (Figures 2E and 3B). Further, we found an enthalpic stabilisation of CSDex folding with increasing concentrations of added DNA or RNA (Figure 5A; Figure S7A, Supporting Information), an indication that nucleic acid binding leads to strengthened intramolecular interactions. Remarkably, unfolding rates (k_u_; Figure 5C,D; Figure S9B, Supporting Information) and the initial D/A (Figure S7B, Supporting Information) decreased significantly with increasing nucleic acid concentrations, consistent with enhanced binding to the native state. These observations are supported by Zhang et.al, who reported that all loops of native CSDex are more compact and rigid in the bound‐DNA (5′ aaCACCt 3′) compared to free conformation.^[^ ^63^ ^]^

The increase of folding stability upon binding with RNA was higher compared to DNA (Figure 3C,F). While most studies report stronger RNA binding than DNA,^[^ ^10^, ^80^, ^88^ ^]^ some suggested the opposite, indicating higher CSD affinity for ssDNA.^[^ ^98^ ^]^ Our result revealed almost the same binding free energies (ΔG_B_^’0^) for both CSDex‐DNA and CSDex‐RNA (Figure 4A–C). Interestingly, CSDex‐RNA binding exhibited a more favorable enthalpy but was associated with a larger entropic penalty compared to CSDex‐DNA binding (Figure 4D). This stronger stabilisation in the presence of RNA could be explained by the two‐fold increase in sugar‐π and π‐π interactions found in protein‐RNA when compared to protein‐DNA complexes.^[^ ^13^ ^]^ Moreover, tighter binding could lead to greater entropic cost for CSDex‐RNA complex formation due to conformational constraint and water structuring.^[^ ^89^, ^90^, ^91^ ^]^ Importantly, our findings demonstrate that binding affinity alone does not fully capture the complexity of CSDex‐DNA/RNA interactions; rather, these interactions appear to be finely tuned to regulate both function and stability.

Furthermore, our results showed that the folding stability is not only type‐dependent (DNA or RNA) but also sequence‐dependent. The stronger increase in CSDex folding stability for specific DNA/RNA sequences (Figure 3F) could be related to the different nucleotides interacting with aromatic groups (Phe, Tyr, Trp, including cationic His) from the protein.^[^ ^11^, ^30^, ^63^, ^87^ ^]^ For instance, we observed an increase in the T_M_ of CSDex upon the addition of DNA in a similar order: CATC > CACC > CAAC ~ ybox (CCAAT) > CAGC (Figure 3F). This could be explained by the increased contact between CSDex Phe, Tyr, and Trp residues and DNA nucleobases in the order T > C > A ≈ G.^[^ ^99^ ^]^ Substituting these aromatic amino acids with alanine showed disruption in the binding with nucleic acids.^[^ ^30^ ^]^ While the impact of sequence on CSD folding stability may differ for longer DNA or RNA,^[^ ^43^ ^]^ the sequence‐dependent π–π interactions with short recognition motifs are expected to remain unchanged.^[^ ^78^ ^]^ Interestingly, YB1 also oligomerises in the presence of longer poly(C), poly(T), or poly(U) ssDNA or ssRNA (≥30 nt), independent of the nucleotide base.^[^ ^77^ ^]^ This oligomerization depends on the CTD domain flanking the CSD, with longer CTD domains preserving mRNA secondary structure, while shorter CTD domains disrupt it.^[^ ^77^ ^]^ Further studies are needed to clarify how longer DNA and RNA sequences modulate CSD folding within the full‐length protein and drive the assembly of active YB1 multimers.^[^ ^77^, ^100^, ^101^ ^]^

All these sequences interacting with CSDex have pivotal cellular functions such as activation or inhibition of translation or transcription, DNA repair, and RNA storage.^[^ ^9^, ^15^, ^102^ ^]^ We show that decreasing cellular RNA content by puromycin or RNase A treatment reduced the folded fraction by ≈7% (Figure 3D,E; Figure S10, Supporting Information). Thus, the interaction of CSDex with DNA or RNA, which significantly increases the folded fraction at physiological conditions (37 °C), could have major biological implications. Particularly, DNA or RNA interaction could work as a conformational switch for CSDex through the regulation of active folded versus inactive unfolded protein. For instance, in response to different stress conditions, YB1 was shown to activate the translation of G3BP1 and consequently promote stress granule assembly.^[^ ^85^ ^]^ Likewise, under arsenite stress, YB1 was shown to induce the translation of HSP70, which is essential to prevent protein aggregation.^[^ ^84^ ^]^ Hence, upon cellular need, the folded fraction of CSDex can be increased by interacting with RNA or DNA, resulting in the expression of the associated protein.

### 3.2 A Mechanistic View of CSDex Interacting with Multiple Nucleic Acids in the Cell

**Figure 6:**
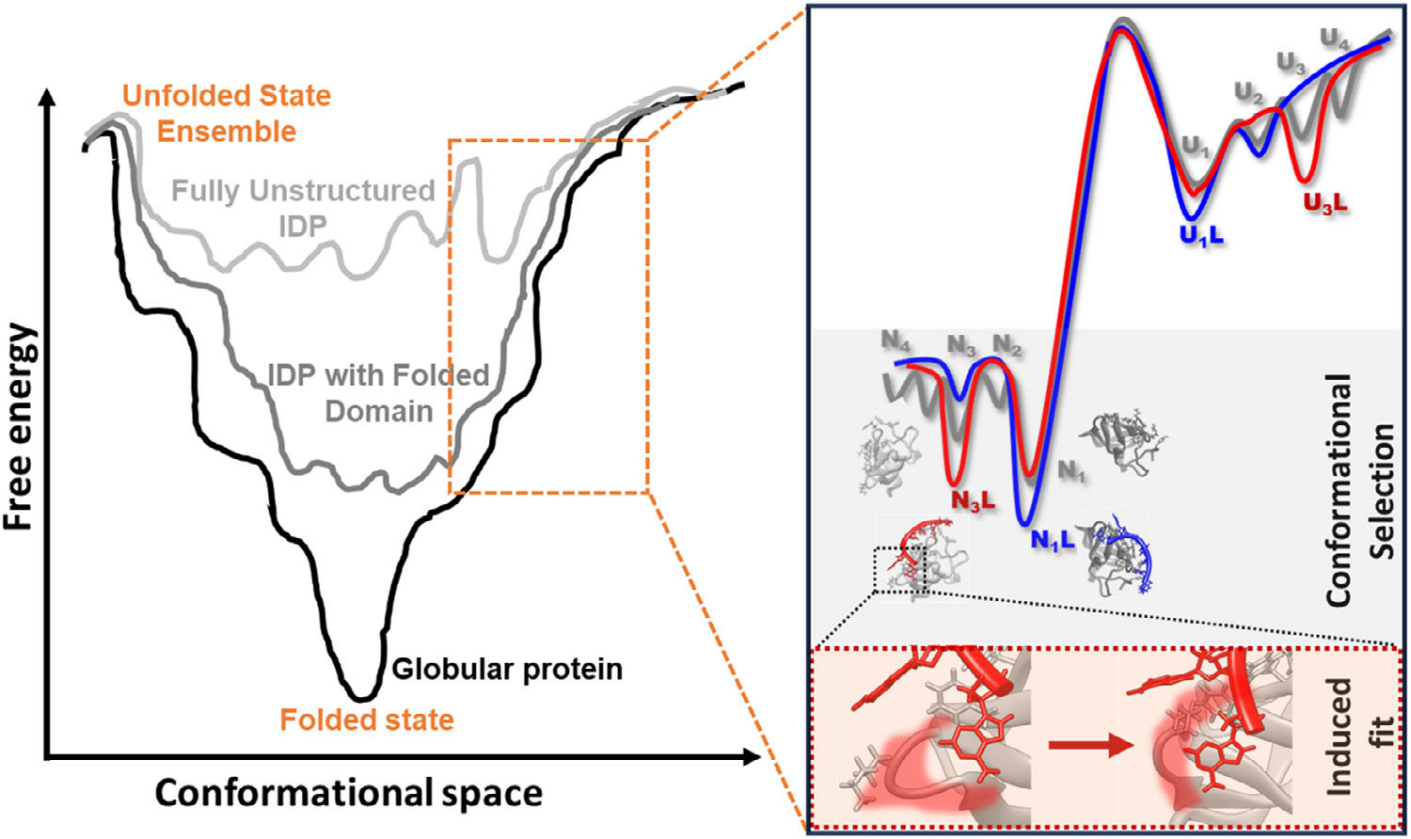
Illustration of the proposed interaction mechanism of CSDex with DNA/RNA. Folding free‐energy landscapes of fully folded protein (black), IDP (light grey), and partially structured IDP (dark grey). YB1 is a partially structured IDP showing a distinct folding transition with a native (N_1_, N_2_, N_3,_ and N_4_) and an unfolded ensemble (U_1_, U_2_, U_3_, and U_4_) of conformational states (inset, grey landscape). Conformational selection due to nucleic acid interaction leads to the stabilization of a distinct state (e.g., N_1_L: RNA binding [blue landscape]; N_3_L: DNA binding [red landscape]), possibly followed by an induced fit of the protein adapting to the ligand. This results in a smoother folding landscape. YB1 structure illustrated by PDB:6LMR.

A mechanistic view of the binding mechanism of CSDex to nucleic acids is required for pharmaceutical targeting of a disease‐relevant protein‐nucleic acid complex. CSDex folding in the absence of nucleic acids involves a complex pathway as suggested by the low ß‐parameters of ≈0.6 to 0.75 over 25 to 41 °C (298–314 K, Figure S11, Supporting Information). Particularly, the low ß‐parameters at conditions where the CSDex is mostly folded (T = 25 °C) indicate that the native state exists as an ensemble of energetically similar conformers (**Figure** **6**, native [N] and native‐like [N_1_, N_2_, N_3_, …]). Remarkably, we observed DNA binding accelerate folding (faster k_f_; Figure 5C,D), while promoting a simple two‐state folding transition as suggested by an increase of ß‐parameter approaching one upon added nucleic acid in vitro (Figure 5B; Figure S9A, Supporting Information). Thus, DNA or RNA binding to the native state promotes a smooth folding pathway by preferentially stabilising a particular native conformer (N → N_1_L, N → N_3_L; Figure 6).^[^ ^61^, ^103^, ^104^, ^105^ ^]^ Preferential binding of DNA or RNA and stabilisation of a particular CSDex native state favours a conformational selection mechanism.^[^ ^58^, ^106^ ^]^ Native‐like conformations could refer to different conformational states of loops 12, 34, and 45, which are flexible and adopt different structures.^[^ ^69^, ^70^ ^]^ Thus, our folding studies together with previous NMR findings of CSDex compaction upon DNA binding^[^ ^63^ ^]^ suggest that DNA or RNA first bind to preexisting folded conformers (conformational selection) and then promote structural adjustments that further stabilize the bound state (induced fit; Figure 6). Despite similar affinities, RNA stabilizes CSDex more than DNA, likely by binding a distinct native state and fine‐tuning a more stable protein‐RNA complex (Figure 6 and N → N_1_L). This is further supported by the distinct secondary structures adopted by CSDex upon RNA versus DNA binding (Figure S6, Supporting Information). Additional evidence emerges from unfolding kinetics, where the rate (k_u_ [37 °C]) with 5 µM RNA (0.08 ± 0.03 s^−1^) is reduced by a factor of two compared to 5 µM DNA (0.14 ± 0.05 s^−1^), reflecting the greater stability of the CSDex‐RNA complex.

## 4 Conclusion

In this work, we showed that the marginal stability of CSD prevails in the cell. Our alanine‐scan mutation analysis showed that the extended disordered C11‐tail (CSDex) increases folding stability through strengthened intramolecular interactions and ligand binding. CSDex is 50% folded and 50% unfolded at physiological conditions, but more folded upon interacting with DNA or RNA. The increase in stability is sequence‐dependent, enabling CSDex to switch between different functional states. For example, YB1 can act as a transcription factor in the nucleus, a translation regulator in the cytoplasm, participate in stress granule formation, and can also work as a nucleic acid chaperone.^[^ ^11^, ^15^, ^77^, ^85^ ^]^ For each function, CSDex is required to adopt a specific native bound conformation, highlighting the importance of a native ensemble with multiple potential binding conformers. In fact, multifunctional proteins like YB1, whose function depends on the ligand type (DNA and RNA), often face higher energy costs and slower conformational changes upon binding when relying solely on an induced fit mechanism. The conformational selection model, which involves a preexisting native ensemble and faster sampling for binding, better explains their functional flexibility.^[^ ^48^, ^107^ ^]^ Hence, the interaction of CSDex‐DNA/RNA is the key to multifunctionality and the reason for enhanced folding stability. Our results suggest that folded but unstable domains in partially structured IDPs could link efficient multiple ligand binding capacity and versatile IDP functionality.

In conclusion, our study has broader implications for further design of specific drugs targeting YB1 in various diseases like cancer and HIV.^[^ ^25^, ^26^, ^82^ ^]^ Targeting YB1 might negatively impact multiple functions or pathways. Nevertheless, each CSDex‐nucleic acid complex may have unique conformations, folding pathways, and energy levels, suggesting that the folding landscape is tuned for ligand interactions. Thus, targeting specific complexes with a certain function holds promise for improving drug development and disease treatment. By targeting certain conformations of folding and binding landscapes, YB1 multifunctionality could be retained by ensuring that other functions remain uncompromised.

## 5 Experimental Section

### Plasmid Construction, Transformation, and Pellet Preparation

Plasmids were prepared by GenScript, where the original CSD sequence from amino acids 52–129 (CSD) and the extended CSD from amino acids 52–140 (CSDex) were subcloned into a modified pDream2.1 vector with an N‐terminal green fluorescent protein (AcGFP1) and a C‐terminal mCherry. Additionally, single mutations (G135A, S136A, and Y138A) were purchased, and the corresponding triple mutant. For the unlabeled CSDex, AcGFP1, and mCherry were replaced by Cysteine (C). The protein sequences for the respective proteins are provided in Table S4 (Supporting Information). Plasmids were transformed into competent E. coli cells (NiCo21 [DE3]; New England Biolabs, C2529H) according to the manufacturer's protocol for protein purification. A single colony was picked and grown to OD 0.6 at 37 °C and 225 rpm, followed by the overexpression induced with 0.1 mM IPTG at 18 °C overnight and 180 rpm. Cells were harvested using centrifugation (5000 × g for 15 min at 4 °C), and the retrieved pellets were stored at −20 °C.

### Protein Purification

The proteins were purified with TALON His‐tag spin columns (Takara) using the manufacturer's protocol. For the mutational study, an equilibration buffer containing 10 mM phosphate‐buffered saline (PBS [Sigma–Aldrich]) with pH 7.4 and an elution buffer containing 1 × PBS + 400 mM Imidazole (Sigma–Aldrich) were used. The purified sample was further dialysed against PBS buffer and concentrated using Amicon ultra 30 kDa centrifugal filters (Millipore). The stability varied from one batch to the other batch, attributed to the ratio of 260 nm/280 nm, which reflects the amount of nucleic acid. The ratio for Y138A was around 1.38; hence, the purified protein contained nucleic acid. An attempt to remove it led to misfolding/aggregation such that unfolding was not evident.

To study the impact of nucleic acid on the folding stability of CSDex, nucleic acid (ssDNA and ssRNA) was purchased from GenScript: ssDNA (aaCATCt, aaCACCt, aaCAACt, gCCAATc, aaCAGCt, aaCCACt, ttttttt); ssRNA (aaCAUCu, aaCACCu, aaCAACu, gCCAAUc, aaCAGCu, aaCCACu, uuuuuuu). The elimination of the nucleic acid during purification was required to observe the effect of additional nucleic acid while avoiding misfolding/aggregation. Therefore, for CSDex, 50 mM phosphate buffer + 500 mM NaCl + 5 mM Mg_2_Cl was used as equilibration buffer, and 10 mM Imidazole and 250 mM Imidazole were added to the wash and elution buffer, respectively, to reduce the nucleic acid amount. It was further dialysed against a PBS buffer. Two batches were purified with a ratio of 0.92 and 1.09. Shock‐freezing in liquid nitrogen and storing at −80 °C led to misfolding. Hence, aliquots of the purified samples were prepared, lyophilised with a SpeedVac at 4 °C, and stored at −20 °C. For all the FReI studies, the sample was purified with a 260 nm/280 nm ratio of 0.92.

For isothermal titration calorimetry (ITC) and circular dichroism (CD) measurements, the purification protocol was optimized to increase the yield of purified FRET‐labeled or unlabeled CSDex. The lysis buffer was supplemented with DNase I and a high salt concentration (1 M NaCl) to reduce nucleic acid content. FRET‐labeled CSDex was purified via His‐tag affinity purification as mentioned above. For the unlabeled CSDex purification, 1 mM DTT was added to all buffers. Following purification, the protein sample was dialyzed against PBS and subsequently lyophilized. For the unlabeled CSDex, a double purification step was undertaken, with His‐tag and analytical size exclusion chromatography (HiLoad Superdex26/600 75 pg column at a flow rate of 0.5 mL min^−1^ at 4 °C). The equilibration buffer contained 1 × PBS and 1 mM DTT.

### Isothermal Titration Calorimetry

To obtain the binding affinities of DNA and RNA to CSDex, ITC was performed on a MicroCal iTC200 by titrating single‐stranded DNA (ssDNA, sequences: aaCACCt and aaCATCt) and single‐stranded RNA (ssRNA, sequences: aaCACCu and aaCAUCu) into the purified FRET‐labeled CSDex. The ITC measurement was conducted at 293 K with a single batch of purified protein (ratio 260 nm/280 nm < 0.6). The thermograms were fitted to obtain the equilibrium dissociation constant (K_d_) using Origin 7 software (MicroCal Inc., USA), assuming a one‐site binding model.

### Circular Dichroism

To determine the melting temperature of the unlabeled CSDex, sequences encoding fluorescent tags at the N‐ and C‐termini were replaced with C from the plasmid. The modified construct was transformed into *E. coli*, and protein expression was carried out under the same conditions as for the FRET‐labeled CSDex. Thermal unfolding of 20 µM of purified protein was performed by gradually increasing the temperature from 20 to 70 °C in 2 °C increments (60 °C/h) using a Chirascan plus CD spectrometer and a quartz cell of 0.1 mm path‐length. At each temperature step, CD spectra were recorded in the wavelength range of 200 to 280 nm. Similar settings were used to measure the FRET‐labeled CSDex. For the reverse scan, the measurements were done from 80 to 20 °C with a 5 °C interval. CD spectra to monitor secondary structure changes upon nucleic acid binding were recorded at 20 °C using 45 µM of CSDex and 20 µM of DNA_CACC_ or RNA_CACC_ in PBS. To calculate the T_M_, the CD signal [ellipticity (θ) was converted, mdeg] at 222 nm was converted to the folded fraction with the following equation (Equation 1) and plotted as a function of the temperature.

(1) $$\mathrm{Folded}\;\mathrm{fraction}\;=\frac{\theta_{U}-\theta }{\theta_{U}-\theta_{F}}$$

By fitting the curve of θ(T) versus T into a two‐state folding model, both T_M_ and van't Hoff enthalpy of unfolding at T_M_ (ΔH_U_^0’^) were obtained (Equation 2), with linear folded(θ_F_ (T) = a_F_  + b_F_T) and unfolded (θ_U_ (T) = a_U_  + b_U_T) baselines.^[^ ^108^, ^109^ ^]^

(2) $$\theta \left(T\right)=\frac{\theta_{F}\left(T\right)+\theta_{U}\left(T\right)\exp\left[-\frac{\Delta H_{U}^{0^{\prime }}}{R\left(\frac{1}{T}-\frac{1}{T_{M}}\right)}\right]}{1+\exp\left[-\frac{\Delta H_{U}^{0^{\prime }}}{R\left(\frac{1}{T}-\frac{1}{T_{M}}\right)}\right]}\;$$

### In‐Cell Measurements

In T25 flasks (Sarstedt), HeLa cells (a kind gift from Rolf Heumann, Ruhr‐Universität Bochum, Germany, contamination‐free) were grown as adherent culture in a humidified atmosphere (37 °C and 10% CO_2_) in DMEM supplemented with 10% FBS, 100 U/mL penicillin, and 0.1 mg/mL streptomycin (all sigma). Upon reaching 80 to 90% confluence, cells were passaged in a 1:4/1:6 ratio every 2 to 3 days using trypsin digestion. For the measurement, transfection was carried out with Lipofectamine 3000 (Thermo Fisher) according to the manufacturer's protocol at 80 to 90% confluence cells on six‐well plates (Sarstedt). Concisely, 2 µg of respective plasmid DNA was mixed with 4 µL of P3000 reagent in 125 µL of Opti‐MEM, which was further mixed with a mixture of 125 µL Opti‐MEM supplemented with 4 µL Lipofectamine 3000 reagent after an incubation time of 10 min. After mixing both and incubating them for 15 min, the transfection mixture was added to the cells and incubated for 6 h in an antibiotic‐free medium. Cells were then passaged using trypsin digestion and seeded on 35 mm glass‐bottom dishes (Fluorodish, WPI), which were further incubated for 48 h with the above‐mentioned cell culture conditions before imaging.

### Cell Treatment with Puromycin and RNase A

Cells were incubated with RNase A (Thermo Scientific) to a final concentration of 5 µg/mL for 1 h, or with puromycin dihydrochloride (MedChemExpress) to 100 µg/mL for 30 min, at 37 °C in a humidified environment containing 5% CO_2_. The buffer used for RNase A storage (used as the vehicle for RNase A) and 0.2% (v/v) DMSO (used as the vehicle for puromycin) were applied to cells under identical conditions. Both control treatments were subjected to the same incubation parameters as their respective experimental conditions. Following incubation, cells were prepared for FReI measurements. The RNase A and puromycin dihydrochloride concentrations were maintained throughout the measurement time.

### Sample Preparation for FReI Measurements

For in vitro FReI measurements: 5 µM samples (purified CSDex or Y138A) were prepared in PBS buffer (with/without additional DNA/RNA). Fifteen microliters of each sample was pipetted between a preprepared glass coverslip (Menzel No. 1.0) with a 120 µm thick imaging spacer (Secure Seal, sigma) and a 35 mm glass bottom dish (Fluorodish, WPI).

For in‐cell FReI measurements: Cells were washed twice with DPBS (Sigma‐Aldrich) to remove dead cells, and this was further placed on a glass coverslip with a 120 µm thick imaging spacer supplied with 30 µL Leibovitz's L15 medium supplemented with 30% FBS.

### FReI Measurements and Data Analysis

Fast relaxation imaging (FReI) was based on Förster resonance energy transfer (FRET) to measure the protein unfolding/folding process induced by rapid laser (IR diode laser: m2K, 2200 nm) heating (millisecond temperature jump) as described previously.^[^ ^62^ ^]^ In short, an IR laser was used to increase the temperature by 2 °C each for 25 s, which was repeated for 10 to 18 jumps, increasing from 20 to 40 to 60 °C, followed by re‐cooling to the initial temperature. The initial temperature varied depending on the constructs from (17–23 °C). The temperature‐sensitive dye Rhodamine B was used for temperature calibration. The intensity profile of a sample of Rhodamine B (100 µM) is shown in Figure S2 (Supporting Information). A polynomial equation from Ross et al.^[^ ^110^ ^]^ was used to determine a ΔT of 2.0 ± 0.14 °C. The fluorescent signal derived by imaging of AcGFP1 (Donor [D]; 497–527 nm) and mCherry (Acceptor [A]; 581–679 nm) emissions acquired by two CCD cameras coupled into a widefield fluorescence microscope provides D and A intensity at a frame rate of two frames per second. Conformational changes due to increased temperature resulted in FRET change (donor‐to‐acceptor fluorescence ratio [D/A]). The ratio of D/A as a function of time was evaluated using ImageJ (US NIH), self‐written Matlab code, and OriginLab pro2024. For the in vitro data: A Region of Interest (ROI) of the same pixel size was drawn on each image of the D and A channels. For the in‐cell data, each cell was separated from the other using a threshold, followed by averaging the cytoplasmic region for each channel individually. The donor and acceptor intensities across the ROI were averaged at each time point, followed by background subtraction for each D and A channel. The D/A ratio was further scaled to (D‐αA; α = D_0_/A_0_) for the unfolding kinetic amplitudes of each temperature and plotted against temperature, which was fitted to single exponentials (Equation 3), representing a two‐state folding behavior with k_obs_ being the observed rate constant for each jump, (𝐴_2_ − 𝐴_0_) being the equilibrium state.

(3) $$D\left(t\right)-\alpha A\left(t\right)=A_{0}+\left(A_{2}-A_{0}\right)\cdot \left(1-\exp^{\left(-k_{\mathrm{obs}}\cdot t\right)}\right)$$

Alternatively, stretched exponential fitting was used for the in vitro experiments for each jump (Equation 4), from which the beta‐parameter (ß), defining the roughness of the folding free‐energy landscape, was determined (ß < 1 indicates a rough energy landscape).

(4) $$D\left(t\right)-\alpha A\left(t\right)=A_{0}+\left(A_{2}-A_{0}\right)\cdot {\left(1-\exp^{\left(-k_{\mathrm{obs}}\cdot t\right)}\right)}^{\beta }$$

In some of the in vitro FReI measurements of CSDex supplemented with the different nucleic acids (ssDNA/ssRNA), slight aggregation at high temperatures was observed (decrease of D/A after unfolding at each T jump). Hence, a linear term was introduced in the stretched exponential fitting (Equation 5) to correct the decrease in the amplitude because of aggregation.

(5) $$D\left(t\right)-\alpha A\left(t\right)=A_{0}+\left(A_{2}-A_{0}\right)\cdot {\left(1-\exp^{\left(-k_{\mathrm{obs}}\cdot t\right)}\right)}^{\beta }+C\left(t\right)$$

The “thermodynamics from kinetics approach” developed by Gruebele and co‐workers^[^ ^64^ ^]^ was applied to determine the melting temperature (T_M_) and modified standard‐state free energies of folding (ΔG_F_^0′^) at 310 K (37 °C; Equation 7).^[^ ^64^ ^]^ Using a two‐state approximation, Girdhar's model (Equation 6) was used to yield T_M_ and cooperativity parameter δg1, where ΔT is the amplitude of each T‐jump (2 °C), A_0_ and m_A_ were the baseline parameters (m_A_ was fixed to 0), R is the universal gas constant (8.314 J mol^−1^ K^−1^).^[^ ^64^ ^]^

(6) $$\begin{array}{ccc} D\left(T\right)-\alpha A\;\left(T\right) & = & \frac{-\delta g_{1}\Delta T\cdot T_{M}}{R{\left(T-\frac{\Delta T}{2}\right)}^{2}}\;\;\cdot \left(A_{0}+m_{A}\left(T-T_{M}\right)\right)\\ & & \cdot \frac{\exp\left[\frac{-\delta g_{1}\left(T\;-\frac{\Delta T}{2}-T_{M}\right)}{R\left(T-\frac{\Delta T}{2}\right)}\right]}{{\left(1+\exp\left[\frac{-\delta g_{1}\left(T\;-\frac{\Delta T}{2}-T_{M}\right)}{R\left(T-\frac{\Delta T}{2}\right)}\right]\right)}^{2}} \end{array}$$

A linear Taylor approximation was used to calculate the modified standard state free energies (ΔG_F_^0’^[37 °C]) via Equation 7, with T = 310 K or 37 °C. This assumes the change in heat capacity for (un)folding (∆C_p_) to be 0.

(7) $$\Delta G_{F}^{0^{\prime }}\left(37^{\circ }C\right)=\delta g1\;*\;\left(T-T_{M}\right)$$

The unfolded (U) or folded (F) fractions, Fraction_i,_ were calculated from the following equation, where “i” = U or F (Equation 8):^[^ ^111^ ^]^

(8) $${\text{Fraction}}_{i}=\frac{1}{1+\exp^{\Delta G_{i}^{0^{\prime }}\left(T\right)/\mathrm{RT}}}$$

The enthalpic (ΔH_F_^0’^) and entropic (ΔS_F_^0’^) components were obtained from the following relation (Equation 9):^[^ ^92^, ^112^ ^]^

(9) $$\Delta G_{F}^{0^{\prime }}\left(T\right)=\Delta H_{F}^{0^{\prime }}\;\;-\left(T*\Delta S_{F}^{0^{\prime }}\right)$$

where ΔH_F_^0′^ stands for modified standard‐state folding enthalpy at T_M_ determined with Equation 10 and ΔS_F_^0′^(T) stands for modified standard‐state folding entropy at T_M_ determined with Equation 11.^[^ ^113^ ^]^

(10) $$\Delta H_{F}^{0^{\prime }}=-\delta g1\;*\;T_{M}$$

(11) $$-T\Delta S_{F}^{0^{\prime }}=-T*\left(-\delta g1\right),\;\mathrm{with}\;T\;=310K\;$$

The protein folding equilibrium could be affected by changes ΔH_F_^0’^ and ΔS_F_^0’^ induced by DNA/RNA binding. The resulting differences in free energy, enthalpy, and entropy could be expressed via ΔΔH_F_^0’^, ΔΔS_F_^0’^, and ΔΔG_F_^0’^as the difference of ΔH_F_^0’^/ΔS_F_^0’^/ΔG_F_^0’^ in the presence of DNA/RNA (ligand condition [LC], with additional DNA/RNA) to the initial stability (buffer), without additional DNA/RNA.

Folding (k_f_) and unfolding (k_u_) rate constants at different temperatures were determined by applying an effective two‐state model and the corresponding relation between the folding equilibrium constant K_F_ and the calculated k_obs_ (k_obs_ = k_f_ + k_u_; Equations 12 and 13):^[^ ^95^ ^]^

(12) $$k_{f}=k_{\mathrm{obs}}*\;{\text{Fraction}}_{F}↔k_{f}=\frac{k_{\mathrm{obs}}*K_{F}}{1+K_{F}}$$

(13) $$k_{u}=k_{\mathrm{obs}}*\;\text{Fractio}n_{U}↔k_{u}=\frac{k_{\mathrm{obs}}}{1+K_{F}}$$

K_F_ (T) was determined by: $\Delta G_{F}^{0^{\prime }}(T)=-R*T*\ln K_{F}\left(T\right)$. The plots describing the temperature dependence of the folding (k_f_) and unfolding (k_u_) rate constants were fitted to an effective two‐state rate model (Equation 14):^[^ ^95^ ^]^

(14) $$\begin{array}{ccc} \ln\left(\frac{k_{0}}{k_{f,u}\left(T\right)}\right) & = & \;\;\frac{\Delta G_{f,u}^{{0^{\prime }}^{‡}}\left(T\right)}{R*T}\\ & = & \frac{1}{R*T}\left[\Delta H_{f,u}^{{0^{\prime }}^{‡}}\left(T_{M}\right)-T*\Delta S_{f,u}^{{0^{\prime }}^{‡}}\left(T_{M}\right)\right.\\ & & +\left.\Delta C_{p;f,u}^{{0^{\prime }}^{‡}}*\left(T-T_{M}+T*\ln\left(\frac{T_{M}}{T}\right)\right)\right] \end{array}$$

with ΔG_f,u_^0’‡^, ΔH_f,u_^0’‡^, ΔS_f,u_^0’‡^ and ΔC_p;f,u_^0’‡^ standing for folding (_f_) or unfolding (_u_) activation free energies, enthalpies, entropies and heat capacities, respectively. When these plots showed no curvature, ΔC_p;f,u_^0’‡^ = 0 was assumed. k_0_ is the rate prefactor, varying with the solvent viscosity (η) according to the relationship (Equation 15):^[^ ^95^ ^]^

(15) $$k_{0}={\left(10\mu s\right)}^{-1}\;*\;{\left(\frac{\eta \left(22{}^{\circ}C\right)}{\eta \left(T\right)}\right)}^{\gamma }$$

(10 µs)^−1^ was used as the upper speed limit for folding.^[^ ^114^ ^]^ The viscosity temperature dependence^[^ ^95^ ^]^ was given by:

(16) $$\eta \left(T\right)=0.226+1.0723*\exp^{\frac{-\left(T-10^{\circ }C\right)}{33}}$$

k_0_ was assumed to vary linearly with the solvent viscosity, with the friction coefficient γ being set to 1.^[^ ^115^ ^]^

### Data Plotting and Statistical Analysis

OriginLab pro2024 was used for graph plotting. Statistical analyses were performed with GraphPad Prism 10.0.1, using a two‐sample t‐test (^***^ *p* < 0.001, ^**^ *p* < 0.01, ^*^ *p* < 0.05) and one‐way ANOVA followed by the Dunnett test for multiple comparisons, computed with a confidence interval of 95%. For all the in vitro measurements, three to four independent replicates were performed, and standard deviation (SD) or the standard error of the mean (SEM) was calculated; for the in‐cell measurements, 25 to 40 cells were treated for each construct. However, single exponential fittings did not converge for all cells, indicating more complex folding pathways than the two‐state. For the analysis, the cells that showed apparent two‐state folding were selected (13–24 cells for each construct) to achieve the thermodynamic parameters (T_M_, ΔG_F_^0’^[37 °C]). Mean values and standard deviations (SDs) or standard errors of the mean (SEMs) for in vitro or in‐cell folding and binding thermodynamic and kinetic parameters, along with statistical analyses, are provided in the Tables S5 to S15 (Supporting Information).

## Conflict of Interest

The authors declare no conflict of interest.

## Supporting information



Supporting Information

## Data Availability

The data that support the findings of this study are available from the corresponding author upon reasonable request.
