# Supplementary material for: Marginal Stability of the YB1 Cold‐Shock Domain in Cells Enables Binding of Multiple Nucleic Acids
Source: Adv Sci (Weinh). 2025 Oct 27;13(1):e12966. doi: 10.1002/advs.202512966 (PMC12767080; doi:10.1002/advs.202512966)
Supplement: Supplementary file 1 — Supporting Information [file ADVS-13-e12966-s001.pdf]

Supporting Information

Marginal Stability of the YB1 Cold-Shock Domain in Cells Enables Binding of Multiple Nucleic Acids

Puja Shrestha[1,2], Sara S. Ribeiro[1,2], Janne Aurich[2], Christian Herrmann[3] and Simon Ebbinghaus[1,2*]

P. Shrestha, S.S. Ribeiro and S. Ebbinghaus
Lehrstuhl für Biophysikalische Chemie and Research Centre Chemical Sciences and Sustainability, Research Alliance Ruhr, Ruhr University Bochum, Universitätsstraße 150, 44801 Bochum, Germany
E-mail: simon.ebbinghaus@rub.de

P. Shrestha, S.S. Ribeiro, J. Aurich and S. Ebbinghaus
Institute of Physical and Theoretical Chemistry, Technical University Braunschweig, Rebenring 56, 38106 Braunschweig, Germany

C. Herrmann
Department of Physical Chemistry I, Ruhr University Bochum, Universitätsstraße 150, 44801 Bochum, Germany

This file contains:
Supplementary Figures S1 to S11
Supplementary Tables S1 to S15

Supplementary figures:

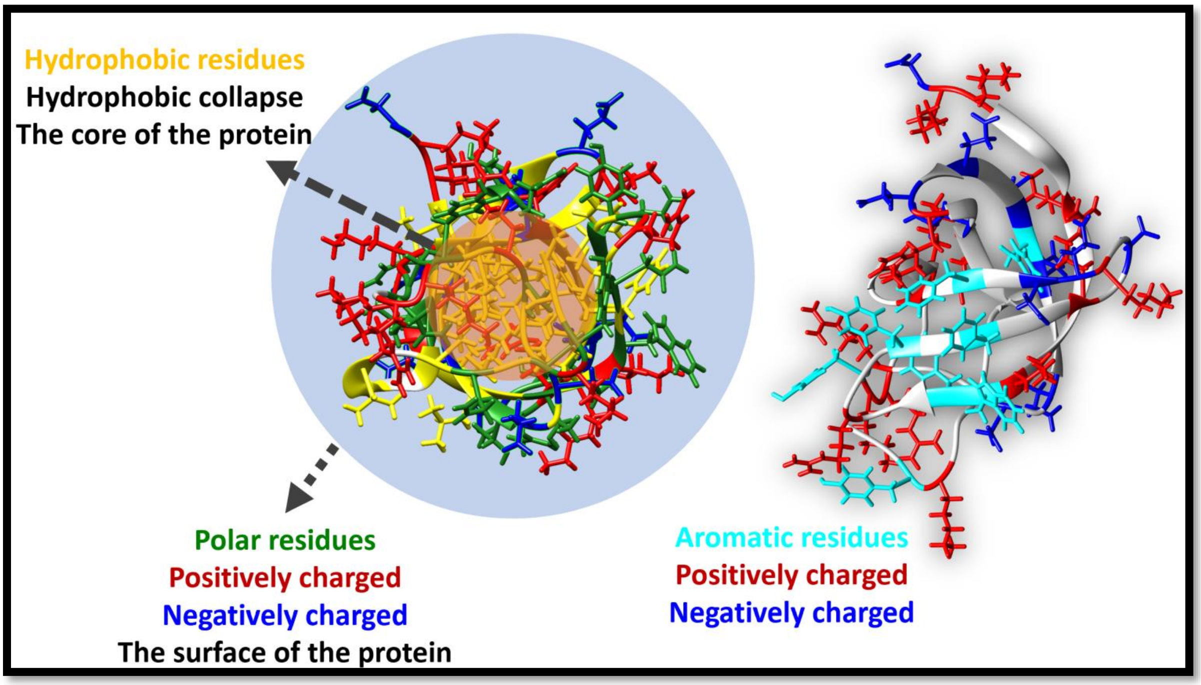

Supplementary figure S1: Structural analysis of CSDex of human YB1 (PDB: 6LMR); done using UCSF Chimera(1): On the left: Top view of the protein: yellow represents the hydrophobic residues, green represents polar residues, red represents positively charged and blue, negatively charged residues. The hydrophobic residues are mostly buried inside the core explained by hydrophobic collapse in protein folding. The outer surface of the protein is mostly polar/charged. On the right: side view of the protein: cyan represents the aromatic residues and red/blues for charged residues. Unlikely other hydrophobic residues, most of the aromatic residues in CSDex are exposed to the surface instead of in the core of the protein, which is energetically unfavourable, as explained by the "icebergs" model suggested by Frank and Evans in 1945 (2).

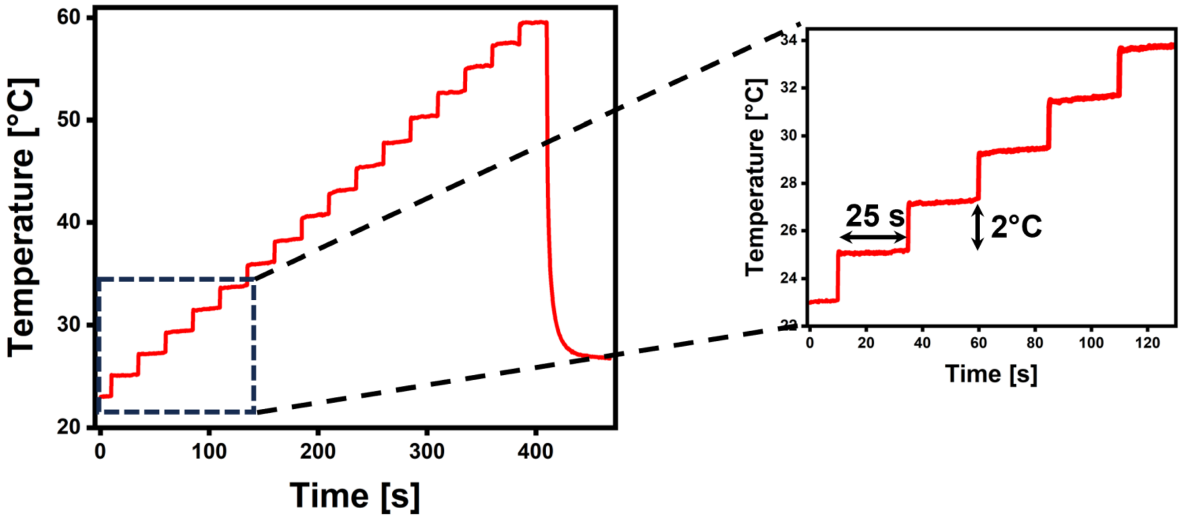

Supplementary figure S2:Temperature profile analysis using Rhodamine B, where each jump resulted in an increase of 2 °C for 25 seconds.

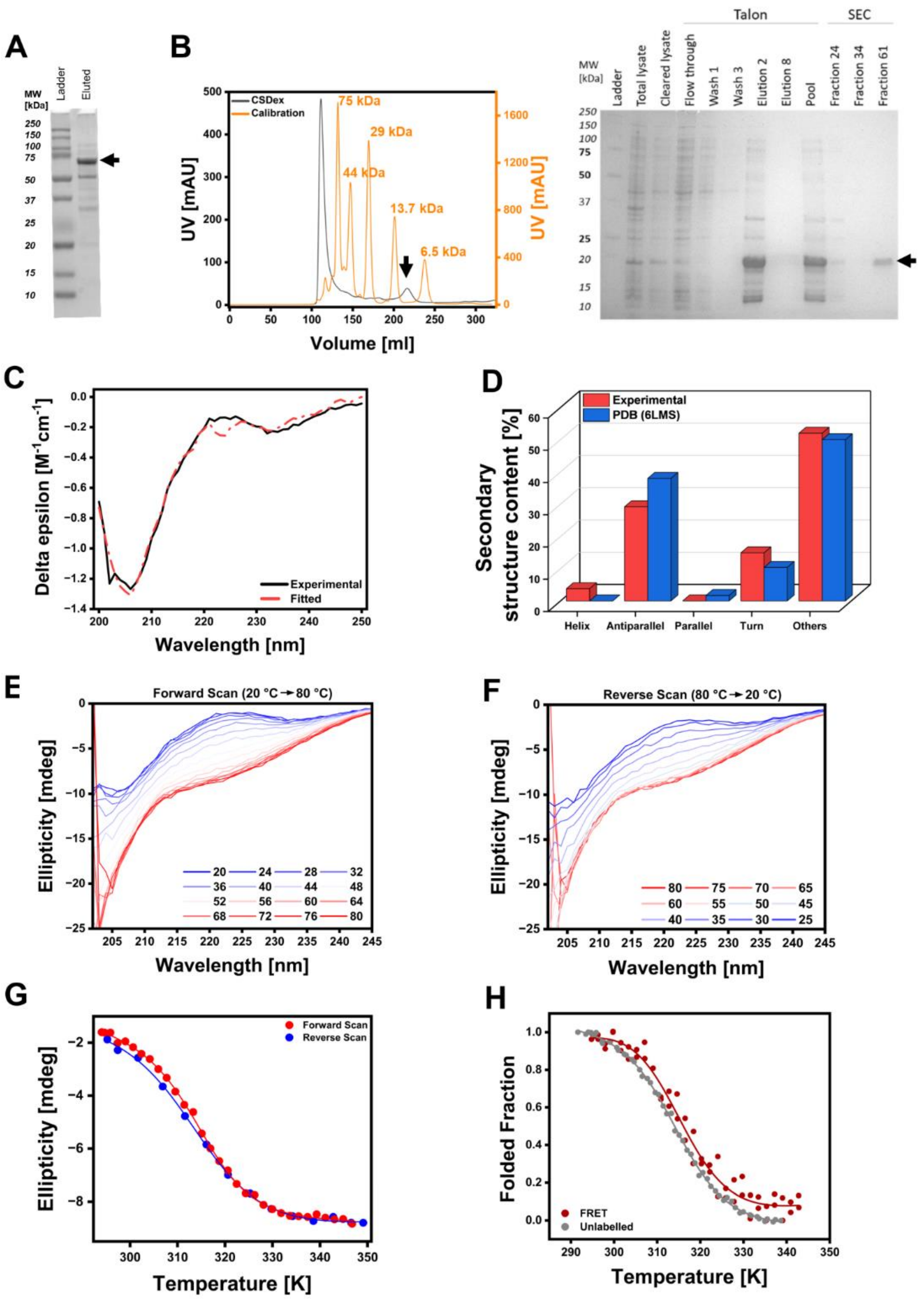

Supplementary figure S3: Folding stabilities of purified unlabelled and FRET-labelled CSDex. (A) SDS-PAGE Gel for purified FRET-labelled-CSDex (~ 66 kDa) (Marker: PageRuler 10-180 kDa; 26616) (B) and unlabelled CSDex (Marker: Precision Plus Protein Dual Color Standards) (CSDex is shown with a black arrow). Size exclusion chromatogram for purified-unlabelled CSDex is shown in (B). Monomeric CSDex molecular weight is ~ 10kDa. C) CD spectra of native CSDex at 20 °C (solid line) and predicted by BeStSel webserver (dashed line). D) Secondary structural content of native CSDex computed by BeStSel from the CD spectra and PDB structure (6LMS), where 'Others' represent the rest of the structural elements and the irregular or disordered structures. E) CD spectra of CSDex showing the transition from native (T = 20°C) to unfolded (T= 80°C) states (Forward scan). F) Reverse scan from 80 °C to 20°C for the reversibility. G) Unlabelled CSDex as a function of temperature retrieved from the ellipticities at 222 nm for both forward and reverse scan. H) Folded fractions of FRET-labelled and unlabelled CSDex as a function of temperature retrieved from the ellipticities at 222 nm.

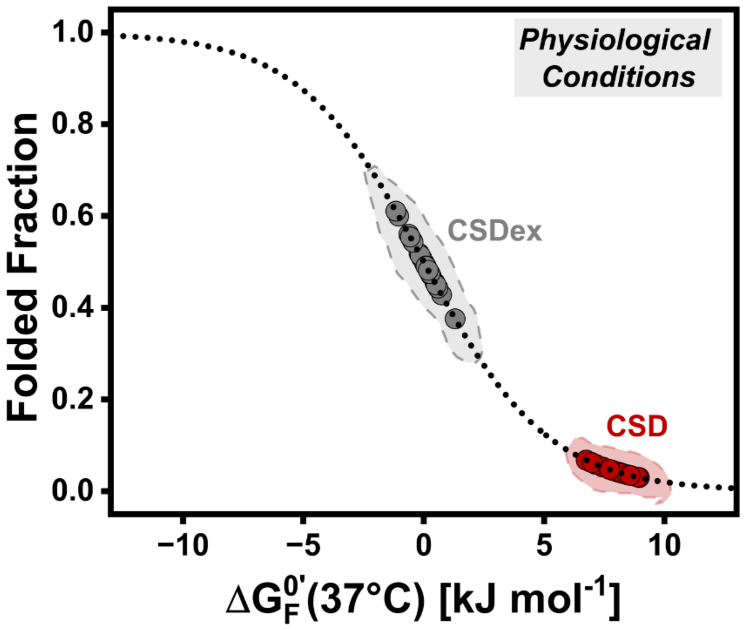

Supplementary figure S4: Modified standard state free-energies of folding ($\Delta G_F^{0'}$(37°C)) and corresponding folded fractions determined for each cell expressing CSD (52-129) and CSDex (52-140). The dot line represents the predicted relationship between folded fraction and $\Delta G_F^{0'}$ for a two-state folding model.

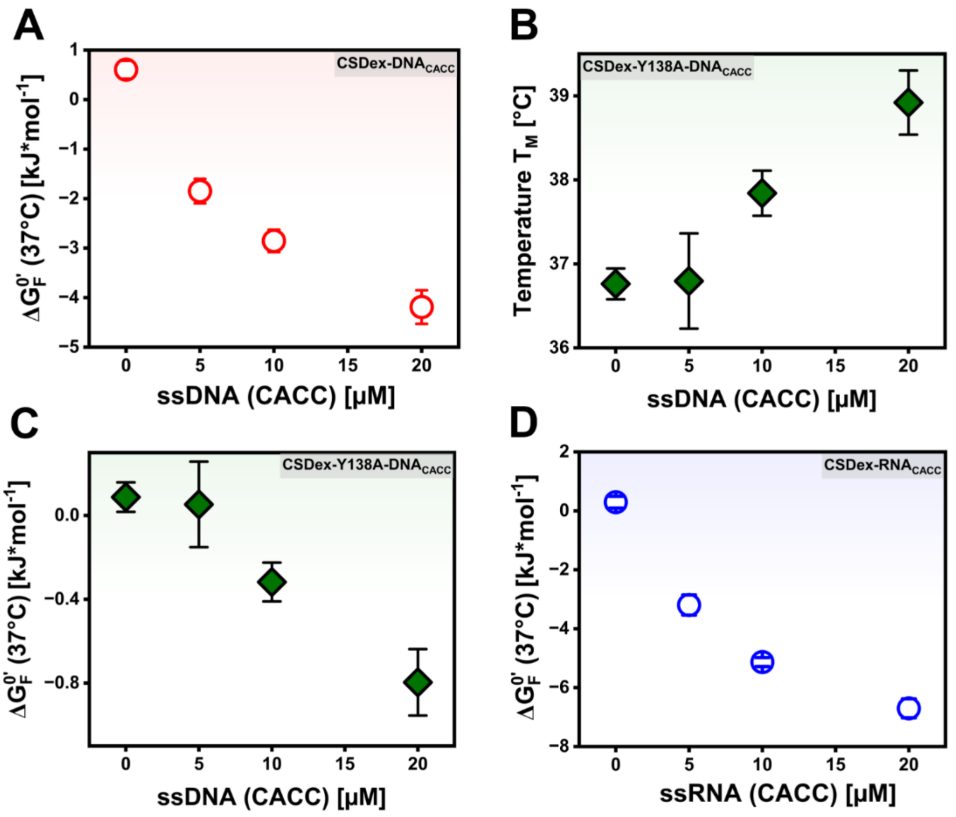

Supplementary figure S5: A) $\Delta G_F^{0'}$(37°C) of CSDex in the presence of ssDNA (CACC with three different concentrations: 5 µM, 10 µM and 20 µM). B) T_M and C) $\Delta G_F^{0'}$(37°C) of CSDex-Y138A in vitro in PBS buffer in the presence of ssDNA with three different concentrations (5 µM, 10 µM and 20 µM). D) $\Delta G_F^{0'}$(37°C) of CSDex in the presence of ssRNA (CACC with three different concentrations: 5 µM, 10 µM and 20 µM).

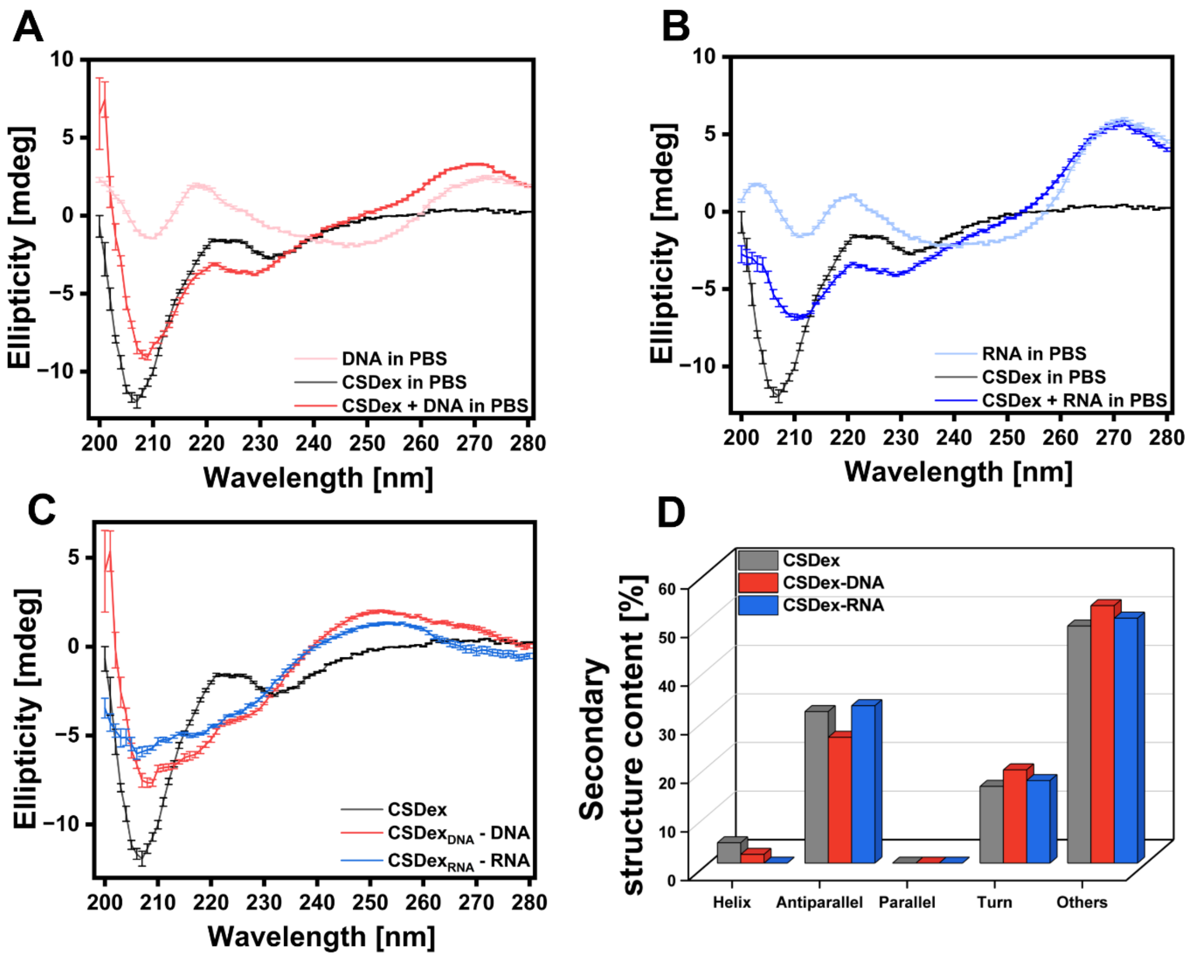

Supplementary figure S6: Secondary structure changes of CSDex upon DNA/RNA binding measured by CD. A) Raw CD – spectra of CSDex, ssDNA and CSDex + ssDNA (CACC) in PBS at 20°C. B) Raw CD – spectra of CSDex, ssRNA and CSDex + ssRNA (CACC) in PBS at 20°C. For (A) and (B): uncertainties are given by standard error of mean (SEM). C) Corrected CD - spectra of CSDex in the presences of DNA or RNA after subtraction of CD – spectra of the respective nucleic acid alone in PBS at 20 °C. Uncertainties are given by propagated SEM. D) Secondary structural content of native CSDex computed by BeStSel from CD – spectra (200 nm -250 nm) presented in (C). 'Others' represent the rest of the structural elements and the irregular or disordered structures.

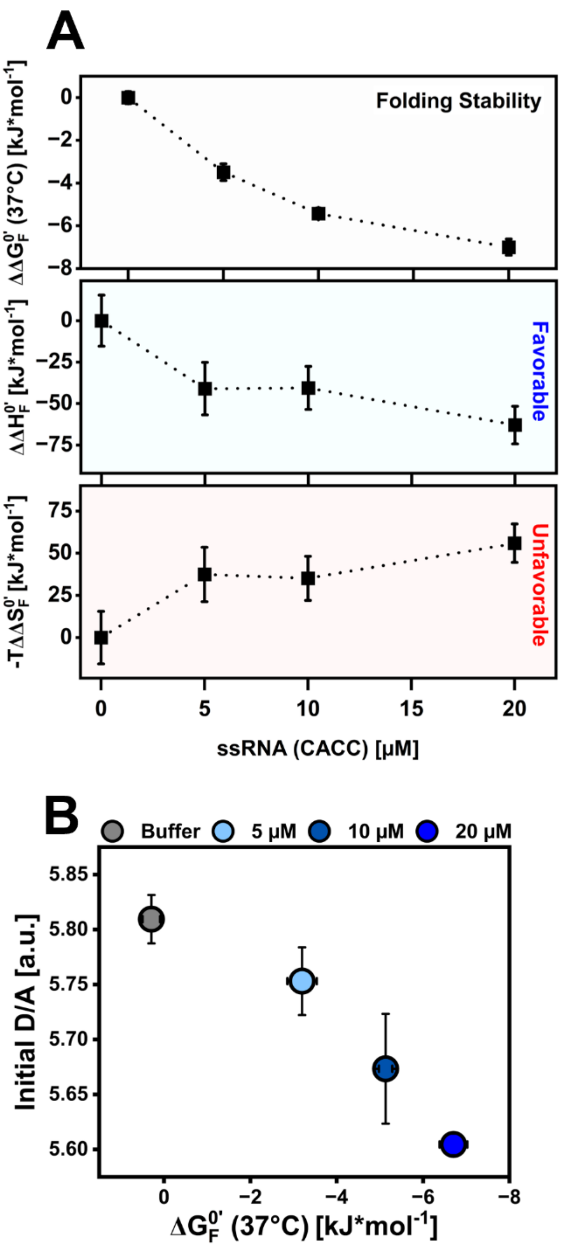

Supplementary figure S7: A) Enthalpic changes ($\Delta\Delta H_F^{0'}$), entropic changes ($-T\Delta\Delta S_F^{0'}$) and $\Delta\Delta G_F^{0'}$(37°C) for CSDex folding in the presence of 5µM, 10 µM and 20 µM ssRNA. B) Initial D/A (at 23 °C) against the $\Delta G_F^{0'}$(37°C) in the presence of additional ssRNA.

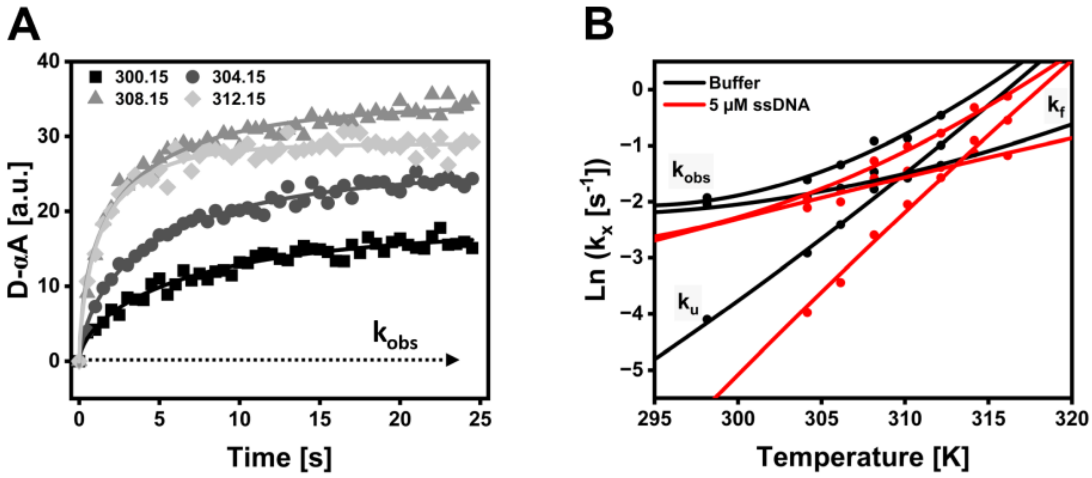

Supplementary figure S8: A) Exemplary relaxation kinetics (D-αA) of CSDex unfolding upon temperature increase in buffer. A stretched exponential model (equation 4) was used to fit the single temperature jumps to determine observed rates constants (k_{obs}). B) Representative rate constants plots (k_{obs}, k_f, k_u) as a function of temperature of CSDex unfolding in the absence and presence of 5 µM ssDNA. Solid lines represent fit to the two-state rate model (equation 14).

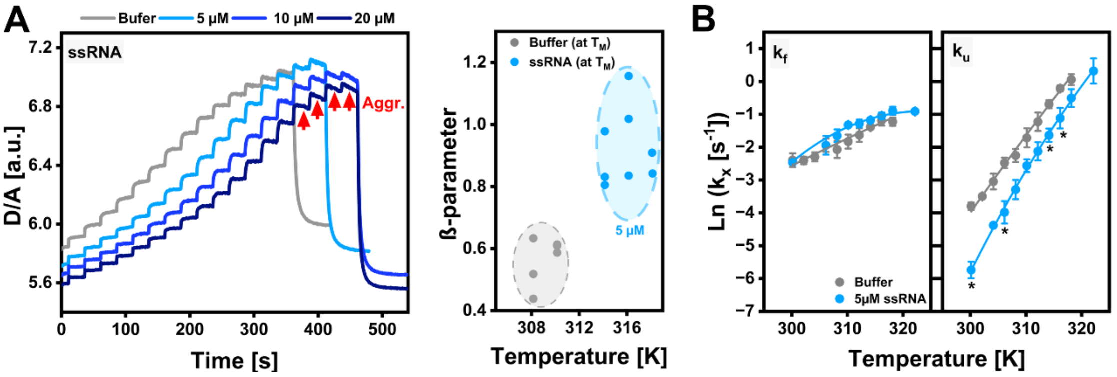

Supplementary figure S9: A) Right: Heat-induced unfolding curve of the D/A of CSDex vs time in PBS buffer in the absence and presence of additional ssRNA (5 µM, 10 µM and 20 µM). Given the propensity of CSDex to aggregate upon addition of 10 µM and 20 µM ssRNA at elevated temperatures, we limited the kinetic analyses to 5 µM RNA. Aggregation behaviour (decline of the D/A curve) pointed by red arrow at higher temperatures. A) Left: ß-parameter for CSDex in buffer condition and CSDex+ssRNA at their corresponding ~ T_M. B) Temperature-dependent folding (k_f) and unfolding (k_u) rate constant plots for CSDex before and after addition of 5 µM ssRNA ($p < 0.05$).*

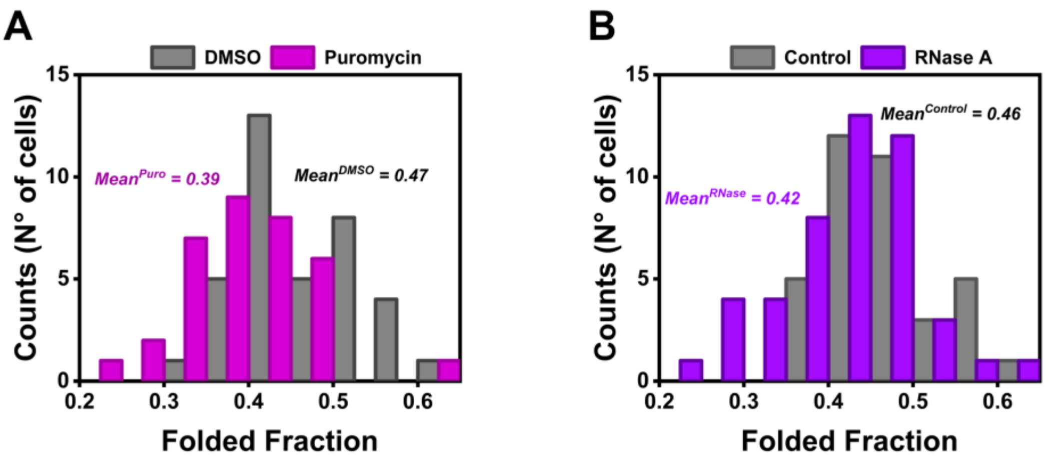

Supplementary figure S10: A and B) Folded fractions histograms for HeLa cells expressing CSDex in physiological (DMSO/control) or treated (puromycin or RNase A) conditions. Mean values assuming a normal distribution for each condition are shown. Folded fractions were calculated using Equation (8).

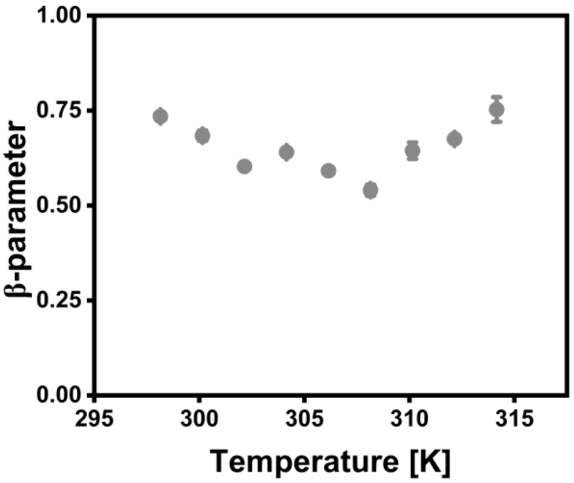

Supplementary figure S11: ß-parameter for CSDex in buffer condition and absence of added nucleic acid at different temperatures (mean ± SEMs).

Supplementary tables:

Supplementary table S1: Thermodynamic parameters for Fret-labelled and unlabelled CSDex derived from CD spectroscopy data. Below: Reversibility data; forward scan is from 20 °C to 80 °C with 2°C increment and reverse scan is from 80°C to 20°C with 5°C interval. Data ± fitting error.

	FRET-Labelled-CSDex			Unlabelled-CSDex		
	**Rep1**	**Rep2**	**Mean ± SD**	**Rep1**	**Rep2**	**Mean ± SD**
**T_M [K]**	317.6 ± 0.8	314.2 ± 0.5	316 ± 2.5	315.9 ± 0.9	314.7 ± 0.9	315.3 ± 0.9
**$\Delta H_u^{0'}$**	100 ± 11	180 ± 20	140 ± 60	150 ± 20	140 ± 20	144 ± 7

	Unlabelled-CSDex	
	**Forward Scan**	**Reverse Scan**
**T_M [K]**	315 ± 3	316 ± 1
**$\Delta H_u^{0'}$**	120 ± 30	150 ± 20

Supplementary table S2: ssDNA and corresponding ssRNA sequences used in this work.

Name	ssDNA	Name	ssRNA
CATC	aaCATCt	CAUC	aaCAUCu
CACC	aaCACCt	CACC	aaCACCu
CAAC	aaCAACt	CAAC	aaCAACu
y-box	gCCAATc	y-box	aCCAAUc
CAGC	aaCAGCt	CAGC	aaCAGCu
CCAC	aaCCACt	CCAC	aaCCACu
7dT	ttttttt	7dU	uuuuuuu

Supplementary table S3: Enthalpy ($\Delta H_{f,u}^{0'\ddagger}$), entropy ($\Delta S_{f,u}^{0'\ddagger}$), and heat capacity ($\Delta C_{p;f,u}^{0'\ddagger}$) of activation for folding(f) and unfolding(u) reactions of CSDex in the absence and presence of ssDNA. The parameters refer to Figure 5D. The fits refer to mean ± SEM of 3 to 4 independent replicates for each condition. The parameters computed from individually fitting of each replicate are shown in Tables S14 and S15.

Folding						
	**$\Delta H_f^{0'\ddagger}$ [kJ mol^{-1}]**	**SE**	**$\Delta S_f^{0'\ddagger}$ [kJ K^{-1} mol^{-1}]**	**SE**	**$\Delta C_{p;f}^{0'\ddagger}$ [kJ K^{-1} mol^{-1}]**	**SE**
**CSDex-buffer**	44.0	5.4	0.02	0.02	0.0	0.0
**CSDex-5 µM DNA**	13.2	9.4	-0.08	0.03	-4.9	2.2
**CSDex-10 µM DNA**	25.8	2.6	-0.04	0.01	-3.9	1.0
**CSDex-20 µM DNA**	22.8	12.5	-0.05	0.04	-7.6	2.7

Unfolding						
	$\Delta H_u^{0'\ddagger}$ [kJ mol^{-1}]	SE	$\Delta S_u^{0'\ddagger}$ [kJ K^{-1} mol^{-1}]	SE	$\Delta C_{p;u}^{0'\ddagger}$ [kJ K^{-1} mol^{-1}]	SE
**CSDex-buffer**	159.0	5.8	0.39	0.02	0.0	0.0
**CSDex-5 µM DNA**	175.7	17.6	0.44	0.06	-1.5	3.0
**CSDex-10 µM DNA**	191.9	5.6	0.49	0.02	-5.9	1.7
**CSDex-20 µM DNA**	154.8	10.3	0.23	0.03	-13.6	3.4

Supplementary table S4: DNA plasmid sequences for FRET labelled CSD/CSDex and unlabelled CSDex.

Name	Plasmid DNA sequence
**Acgfp1-CSD_mCherry**	MVSKGAELFTGIVPILIELNGDVNGHKFSVSGEGEGDATYGKLTLKFIC<br>TTGKLPVPWPTLVTTLSYGVQCFSRYPDHMKQHDFFKSAMPEGYIQER<br>TIFFEDDGNYKSRAEVKFEGDTLVNRIELTGTDFKEDGNILGNKMEYN<br>YNAHNVYIMTDKAKNGIKVNFKIRHNIEDGSVQLADHYQQNTPIGDGP<br>VLLPDNHYLSTQSALSKDPNEKRDHMIYFGFVTAAAITHGMDELYKDI<br>KKVIATKVLGTVKWFNVRNGYGFINRNDTKEDVFVHQTAIKKNNPRK<br>YLRSVGDGETVEFDVVEGEKGAEAANVTGPGKLMVSKGEEDNMAIIK<br>EFMRFKVHMEGSVNGHEFEIEGEGEGRPYEGTQTAKLKVTKGGPLPFA<br>WDILSPQFMYGSKAYVKHPADIPDYLKLSFPEGFKWERVMNFEDGGV<br>VTVTQDSSLQDGEFIYKVKLRGTNFPSDGPVMQKKTMGWEASSERMY<br>PEDGALKGEIKQRLKLKDGGHYDAEVKTTYKAKKPVQLPGAYNVNIK<br>LDITSHNEDYTIVEQYERAEGRHSTGGMDELYK
**Acgfp1-CSDex_mCherry**	MVSKGAELFTGIVPILIELNGDVNGHKFSVSGEGEGDATYGKLTLKFIC<br>TTGKLPVPWPTLVTTLSYGVQCFSRYPDHMKQHDFFKSAMPEGYIQER<br>TIFFEDDGNYKSRAEVKFEGDTLVNRIELTGTDFKEDGNILGNKMEYN<br>YNAHNVYIMTDKAKNGIKVNFKIRHNIEDGSVQLADHYQQNTPIGDGP<br>VLLPDNHYLSTQSALSKDPNEKRDHMIYFGFVTAAAITHGMDELYKDI<br>KKVIATKVLGTVKWFNVRNGYGFINRNDTKEDVFVHQTAIKKNNPRK<br>YLRSVGDGETVEFDVVEGEKGAEAANVTGPGGVPVQGSKYAAKLMV<br>SKGEEDNMAIIKEFMRFKVHMEGSVNGHEFEIEGEGEGRPYEGTQTAK<br>LKVTKGGPLPFAWDILSPQFMYGSKAYVKHPADIPDYLKLSFPEGFKW<br>ERVMNFEDGGVVTVTQDSSLQDGEFIYKVKLRGTNFPSDGPVMQKKT<br>MGWEASSERMYPEDGALKGEIKQRLKLKDGGHYDAEVKTTYKAKKP<br>VQLPGAYNVNIKLDITSHNEDYTIVEQYERAEGRHSTGGMDELYK
**C-CSDex-C**	CKKVIATKVLGTVKWFNVRNGYGFINRNDTKEDVFVHQTAIKKNNPR<br>KYLRSVGDGETVEFDVVEGEKGAEAANVTGPGGVPVQGSKYAAC

*Supplementary table S5:Number of cells for CSD/CSDex Wt and each mutation; its $\Delta G_F^{0'}$(37°C) and T_M (Fig. 2A). The measurements were conducted at 3-4 biological replicates. Significant differences between data sets were tested using One-way ANOVA and the Dunnett test for multiple comparisons (***p < 0.001, **p< 0.01, *p < 0.05).*

$\Delta G^{0'}_F$ (37°C) [kJ mol^{-1}]						
	**N total**	**Mean**	**SD**	**SEM**	**p-value considering CSDex as a control group**	**p-value considering CSD as a control group**
**CSD (129)**	17	8.0	0.7	0.2	<0.001	-
**Triple mutant**	13	6.1	1.3	0.4	<0.001	<0.001
**G135A**	18	4.9	1.1	0.3	<0.001	<0.001
**S136A**	21	1.0	0.4	0.1	.001	<0.001
**Y138A**	22	1.8	0.8	0.2	<0.001	<0.001
**CSDex (140)**	24	-0.02	0.58	0.12	-	<0.001
**Melting temperature T_M [°C]**						
	**N total**	**Mean**	**SD**	**SEM**	**p-value considering CSDex as a control group**	**p-value considering CSD as a control group**
**CSD (129)**	17	26.9	1.6	0.4	<0.001	-
**Triple mutant**	13	29.0	2.5	0.7	<0.001	<0.001
**G135A**	18	30.3	1.1	0.3	<0.001	<0.001
**S136A**	21	35.5	0.6	0.1	<0.001	<0.001
**Y138A**	22	34.2	1.4	0.3	<0.001	<0.001
**CSDex (140)**	24	37.1	0.9	0.2	-	<0.001

*Supplementary table S6: Number of replicas for Buffer/control (without additional ssDNA) and ligand condition (LC with additional ssDNA); its T_M (Figs. 2E & F). Significant differences between data sets were tested using One-way ANOVA and the Dunnett test for multiple comparisons considering corresponding buffer/buffer Y138A as a control group (***p < 0.001, **p< 0.01, *p < 0.05). Table below: thermodynamic parameters (Fig. 5A)*

**CSDex+ssDNA**	**N total**	**T_M [°C] Mean**	**SD**	**SEM**	**p-value considering C1 as a control group**
**CSDex-Buffer (0 µM $_{CACC}$)**	3	35.3	0.8	0.5	-
**CSDex-5µM DNA$_{CACC}$**	4	41.0	1.3	0.7	0.001
**CSDex-10µM DNA$_{CACC}$**	4	42.2	1.0	0.5	<0.0001
**CSDex-20µM DNA$_{CACC}$**	4	44.2	1.4	0.7	<0.0001

**CSDexY138A+ssDNA**	**N total**	**T_M [°C] Mean**	**SD**	**SEM**	**p-value considering Y138A$_{Buffer}$ as a control group**
**CSDex-Y138A-Buffer (0 µM $_{CACC}$)**	5	36.8	0.4	0.2	-
**CSDex-Y138A-5µM DNA$_{CACC}$**	5	36.8	1.3	0.6	0.9998
**CSDex-Y138A-10µM DNA$_{CACC}$**	5	37.8	0.6	0.3	0.1455
**CSDex-Y138A-20µM DNA$_{CACC}$**	5	38.9	0.8	0.4	0.0026

	$\Delta H^{0'}_F$ [kJ/mol]			$-T*\Delta S^{0'}_F$ [kJ/mol]			$\Delta G^{0'}_F$ [kJ/mol]		
**CSDex+ssDNA**	**Mean**	**SD**	**SEM**	**Mean**	**SD**	**SEM**	**Mean**	**SD**	**SEM**
**CSDex-Buffer (0 µM$_{CACC}$)**	-114	21	12	115	21	12	0.6	0.3	0.2
**CSDex-5µM DNA$_{CACC}$**	-150	8	5	148	10	6	-1.9	0.5	0.3
**CSDex-10µM DNA$_{CACC}$**	-175	8	4	172	8	4	-2.9	0.5	0.2
**CSDex-20µM DNA$_{CACC}$**	-186	8	4	182	8	4	-4.2	0.7	0.3

*Supplementary table S7: Number of replicas for Buffer (without additional ssRNA) and ligand condition (LC with additional ssRNA); its T_M (Fig. 3B). Significant differences between data sets were tested using One-way ANOVA and the Dunnett test for multiple comparisons considering C2 as a control group (***$p < 0.001$, **$p < 0.01$, *$p < 0.05$). Table below: thermodynamic parameters (Fig. S7)*

**CSDex+ssRNA**	**N total**	**T_M [°C] Mean**	**SD**	**SEM**	**p-value considering C2 as a control group**
**CSDex-Buffer (0 µM $_{CACC}$)**	3	36.3	0.9	0.5	-
**CSDex-5µM RNA$_{CACC}$**	3	43.6	1.9	1.1	0.0001
**CSDex-10µM RNA$_{CACC}$**	4	47.5	1.1	0.5	<0.0001
**CSDex-20µM RNA$_{CACC}$**	3	49.0	0.9	0.5	<0.0001

	$\Delta H^{0'}_F$ [kJ/mol]			$-T*\Delta S^{0'}_F$ [kJ/mol]			$\Delta G^{0'}_F$ [kJ/mol]		
**CSDex+ssRNA**	**Mean**	**SD**	**SEM**	**Mean**	**SD**	**SEM**	**Mean**	**SD**	**SEM**
**CSDex-Buffer (0 µM $_{CACC}$)**	-117	19	11	118	19	11	0.3	0.4	0.2
**CSDex-5µM RNA$_{CACC}$**	-158	20	12	155	21	12	-3.2	0.6	0.3
**CSDex-10µM RNA$_{CACC}$**	-158	14	7	153	14	7	-5.1	0.3	0.2
**CSDex-20µM RNA$_{CACC}$**	-180	5	3	174	5	3	-6.7	0.6	0.3

*Supplementary table S8: Number of replicas for Buffer/control (without additional ssDNA) and ligand condition (LC with additional ssDNA); its ΔT_M (Fig. 3F). Significant differences between data sets were tested using One-way ANOVA and the Dunnett test for multiple comparisons considering C4 and C5 as a control group (***$p < 0.001$, **$p < 0.01$, *$p < 0.05$).*

**CSDex+ssDNA**	**N total**	**T_M [°C] Mean**	**SD**	**SEM**	**p-value considering C4 as a control group**	**p-value considering C5 as a control group**
**Control 4 (C4)**	4	35.5	1.7	0.9	-	-
**Control 5 (C5)**	4	35.3	0.6	0.3	-	-
**CAAC**	3	36.8	0.6	0.3	0.3225	-
**CATC**	4	42.6	0.7	0.4	<0.0001	-
**Ybox**	3	37.9	2.5	1.5	-	0.061
**CAGC**	3	36.2	1.1	0.6	-	0.779
**CCAC**	3	36.4	0.9	0.5	-	0.613
**7dt**	4	36.2	0.6	0.3	-	0.740

*Supplementary table S9: Number of replicas for Buffer/control (without additional ssRNA) and ligand condition (LC with additional ssRNA); its ΔT_M (Fig. 3F). Significant differences between data sets were tested using One-way ANOVA and the Dunnett test for multiple comparisons considering C1, C2 and C3 as a control group (***p < 0.001, **p< 0.01, *p < 0.05).*

CSDex+ssRNA	N total	T_M [°C] Mean	SD	SEM	p-value considering C1 as a control group	p-value considering C2 as a control group	p-value considering C3 as a control group
**Control 1 (C1)**	3	35.3	0.8	0.5	-	-	-
**Control 2 (C2)**	3	36.3	0.9	0.5	-	-	-
**Control 3 (C3)**	3	37.7	0.2	0.1	-	-	-
**CAAC**	4	43.9	0.5	0.2	-	-	<0.0001
**CAUC**	4	48.1	1.5	0.8	-	-	<0.0001
**Ybox**	4	40.1	1.2	0.6	-	0.002	-
**CAGC**	4	45.0	0.5	0.2	-	-	<0.0001
**CCAC**	3	39.3	1.6	0.9	0.020	-	-
**7du**	3	36.7	0.7	0.4	-	0.94	-

Supplementary table S10: One-site binding model–fitted parameters for the DNA and RNA titration measurements, along with corresponding fitting errors.

Name	N	error	$\Delta H^{0'}_B$ [kcal/mol]	error	$\Delta S^{0'}_B$ [cal/mol/deg]	K_a [mol^{-1}]	error
**1CSDex-DNA$_{CACC}$**	0.99	0.01	-36.2	0.5	-93	5.7E+06	9E+05
**2CSDex-DNA$_{CACC}$**	0.91	0.01	-31.9	0.2	-79	3.8E+06	3E+05
**3CSDex-DNA$_{CACC}$**	0.80	0.01	-37.2	0.4	-97	3.0E+06	3E+05
**CSDex-DNA$_{CATC}$**	0.88	0.01	-29.6	0.3	-69	9.7E+06	1E+06
**1CSDex-RNA$_{CACC}$**	0.77	0.01	-44.8	0.6	-124	1.7E+06	2E+05
**2CSDex-RNA$_{CACC}$**	0.89	0.01	-48.6	0.7	-136	3.3E+06	4E+05
**CSDex-RNA$_{CAUC}$**	0.90	0.01	-38.2	0.2	-98	1.2E+07	9E+05

Supplementary table S11: Thermodynamic parameters derived from ITC data for DNA and RNA titration measurement. Converted to kJ/mol from table S10.

Name	$\Delta H^{0'}_B$ [kJ/mol]	fitting error	$\Delta S^{0'}_B$ [kJ/mol*K]	$-T*\Delta S^{0'}_B$ [kJ/mol]	$\Delta G^{0'}_B$ [kJ/mol]	error
**1CSDex-DNA$_{CACC}$**	-151	2	-0.4	114	-38	2
**2CSDex-DNA$_{CACC}$**	-134	1	-0.3	97	-37	1
**3CSDex-DNA$_{CACC}$**	-156	2	-0.4	119	-36	2
**CSDex-DNA$_{CATC}$**	-124	1	-0.3	85	-39	1
**1CSDex_RNA$_{CACC}$**	-188	2	-0.5	152	-35	2
**2CSDex-RNA$_{CACC}$**	-204	3	-0.6	167	-37	3
**CSDex-RNA$_{CAUC}$**	-160	1	-0.4	120	-40	1

Supplementary table S12: Mean ± SDs for determined (un)folding kinetic parameters (k_{obs}, k_f, k_u) at 37 °C of CSDex in the absence and presence of 5, 10 and 20 µM ssDNA (CACC).

Name	k_{obs} (s^{-1})	SD	k_f (s^{-1})	SD	k_u (s^{-1})	SD
**CSDex-buffer**	0.32	0.10	0.14	0.05	0.18	0.06
**CSDex-5µM DNA_{CACC}**	0.43	0.12	0.29	0.08	0.14	0.05
**CSDex-10µM DNA_{CACC}**	0.34	0.12	0.25	0.08	0.08	0.04
**CSDex-20µM DNA_{CACC}**	0.26	0.06	0.22	0.04	0.05	0.02

Supplementary table S13: Mean ± SDs for determined (un)folding kinetic parameters (k_{obs}, k_f, k_u) at 37 °C of CSDex in the absence and presence of 5 µM ssRNA (CACC).

Name	k_{obs} (s^{-1})	SD	k_f (s^{-1})	SD	k_u (s^{-1})	SD
**CSDex-buffer**	0.36	0.15	0.17	0.06	0.19	0.09
**CSDex-5µM RNA_{CACC}**	0.35	0.06	0.27	0.03	0.08	0.03

Supplementary table S14:Enthalpy ($\Delta H_f^{0'\ddagger}$), entropy ($\Delta S_f^{0'\ddagger}$), and heat capacity ($\Delta C_{p;f}^{0'\ddagger}$) of activation for folding(f) of CSDex in the absence and presence of ssDNA. The fits refer to individually fitting of each replicate. SE stands for standard error or fitting error.

Folding							
**Name**	**$\Delta H_f^{0'\ddagger}$ [kJ mol^{-1}]**	**SE**	**$\Delta S_f^{0'\ddagger}$ [kJ K^{-1} mol^{-1}]**	**SE**	**$\Delta C_{p;f}^{0'\ddagger}$ [kJ K^{-1} mol^{-1}]**	**SE**	**R^2_f**
**Buffer-rep1**	42	5	0.01	0.02	0	0	0.907
**Buffer-rep2**	42	8	0.01	0.02	0	0	0.860
**Buffer-rep3**	50	15	0.04	0.05	0	0	0.783
**CSDex-5µM DNA**	59	6	0.07	0.02	0	0	0.941
**CSDex-5µM DNA**	45	6	0.03	0.02	0	0	0.893
**CSDex-5µM DNA**	33	11	-0.01	0.04	0	0	0.965
**CSDex-5µM DNA**	52	17	0.05	0.05	0	0	0.619
**CSDex-10µM DNA**	10	7	-0.08	0.02	0	0	0.290
**CSDex-10µM DNA**	53	7	0.05	0.02	0	0	0.898
**CSDex-10µM DNA**	46	15	0.03	0.05	-5	4	0.943
**CSDex-10µM DNA**	-6	11	-0.14	0.04	-11	2	0.964
**CSDex-20µM DNA**	19	12	-0.06	0.04	-9	2	0.983
**CSDex-20µM DNA**	-7	21	-0.14	0.07	-16	5	0.888
**CSDex-20µM DNA**	-1	12	-0.12	0.04	-11	3	0.946
**CSDex-20µM DNA**	-31	18	-0.21	0.06	-29	5	0.965

Supplementary table S15: Enthalpy ($\Delta H_u^{0'\ddagger}$), entropy ($\Delta S_u^{0'\ddagger}$), and heat capacity ($\Delta C_{p;u}^{0'\ddagger}$) of activation for unfolding(u)of CSDex in the absence and presence of ssDNA. The fits refer to individually fitting of each replicate. SE stands for standard error or fitting error.

**Unfolding**							
**Name**	**$\Delta H_u^{0'\ddagger}$ [kJ mol^{-1}]**	**SE**	**$\Delta S_u^{0'\ddagger}$ [kJ K^{-1} mol^{-1}]**	**SE**	**$\Delta C_{p;u}^{0'\ddagger}$ [kJ K^{-1} mol^{-1}]**	**SE**	**R^2_u**
**Buffer-rep1**	176	5	0.45	0.02	0	0	0.994
**Buffer-rep2**	158	8	0.39	0.02	0	0	0.989
**Buffer-rep3**	141	15	0.33	0.05	0	0	0.967
**CSDex-5µM DNA**	216	6	0.57	0.02	0	0	0.995
**CSDex-5µM DNA**	185	6	0.47	0.02	0	0	0.993
**CSDex-5µM DNA**	187	11	0.48	0.04	0	0	0.997
**CSDex-5µM DNA**	188	17	0.48	0.05	0	0	0.956
**CSDex-10µM DNA**	187	7	0.48	0.02	0	0	0.993
**CSDex-10µM DNA**	230	7	0.61	0.02	0	0	0.994
**CSDex-10µM DNA**	209	15	0.54	0.05	-5	4	0.995
**CSDex-10µM DNA**	175	11	0.44	0.04	-11	2	0.998
**CSDex-20µM DNA**	201	12	0.52	0.04	-9	2	0.999
**CSDex-20µM DNA**	173	21	0.43	0.07	-16	5	0.991
**CSDex-20µM DNA**	196	12	0.50	0.04	-11	3	0.998
**CSDex-20µM DNA**	154	18	0.37	0.06	-29	5	0.997

References

1. Pettersen,E.F., Goddard,T.D., Huang,C.C., Couch,G.S., Greenblatt,D.M., Meng,E.C. and Ferrin,T.E. (2004) UCSF Chimera--a visualization system for exploratory research and analysis. *J Comput Chem*, **25**, 1605–1612.

2. Frank,H.S. and Evans,M.W. (1945) Free Volume and Entropy in Condensed Systems III. Entropy in Binary Liquid Mixtures; Partial Molal Entropy in Dilute Solutions; Structure and Thermodynamics in Aqueous Electrolytes. *The Journal of Chemical Physics*, **13**, 507–532.
